# Anisotropic boundary-aware detection for cotton leaf diseases with boundary-decoupled regression and lightweight feature adaptation

**DOI:** 10.3389/fpls.2026.1848014

**Published:** 2026-07-15

**Authors:** Bingyu Cao, Zhikai Yang, Wei Chen, Wei Wang, Yutian Yang, Mingqi Kan, Yingchao Wang, Peng Zhou

**Affiliations:** 1 School of Information Science and Engineering, Xinjiang College of Science & Technology, Korla, Xinjiang, China; 2 School of Artificial Intelligence and Data Science, Hebei University of Technology, Tianjin, China; 3 School of Artificial Intelligence, YanShan University, Qinhuangdao, Hebei, China; 4 Yuli Lihua Modern Agriculture Company Limited, Korla, Xinjiang, China; 5 Xinjiang Lihua (Group) Co., Ltd., Korla, Xinjiang, China

**Keywords:** agricultural computer vision, anisotropic feature learning, boundary-decoupled regression, cotton leaf disease detection, edge deployment

## Abstract

Detecting cotton leaf diseases in open-field environments is challenging due to cluttered backgrounds, scale variation, and irregular lesion morphology. Conventional detectors rely on isotropic receptive fields and coupled box-regression losses, which limit their ability to localize elongated lesions with poorly defined boundaries. We present an anisotropic boundary-aware detection framework that propagates high-frequency boundary information across four successive pipeline stages. In the backbone, an Anisotropic Morphological Contrast Aggregation module (AMCA) enhances direction-aware representation and lesion–background contrast via re-parameterizable strip convolutions and high-frequency residual extraction. A Dynamic Semantic Boundary Transfer mechanism (DSBT) then captures boundary priors from shallow layers before they are lost to downsampling and injects them into the neck. A Morphological–Spectral Synergistic Feature Pyramid Network (MFS-FPN) preserves these cues during multi-scale fusion through spatial-domain operations compatible with edge hardware. Finally, an Anisotropic Boundary-Decoupled IoU loss (ABD-IoU) independently penalizes each of the four box boundaries and sustains optimization signals in high-IoU regimes via a logarithmic modulation factor. On the self-constructed Complex Cotton Leaf Disease dataset (CCLD; 6,856 images, 6 classes), the method achieves 78.50% mAP@50 and 65.00% mAP@50:95, improving the YOLOv11n baseline by 4.80% and 2.70% with only 2.73 M parameters at 202 FPS. Cross-domain evaluations on PlantDoc and RWD confirm consistent improvements. The framework runs in real time on NVIDIA Jetson edge platforms with INT8 quantization.

## 1 Introduction

Cotton (Gossypium spp.) is one of the most important natural-fiber crops worldwide, and its health directly affects both yield and the quality of the textile supply chain. During the growth cycle, Verticillium wilt (Verticillium dahliae), Fusarium wilt (Fusarium oxysporum), and various leaf-spot diseases are the principal biotic stresses. Verticillium wilt alone can cause yield losses of 10%–35% across cultivars and growing regions (Gui et al., 2025). Manual scouting over large cotton fields is inherently slow and subjective, making it difficult to meet the time-critical demands of early disease identification (Shafay et al., 2025). Single-stage detectors such as the YOLO family offer a practical balance between inference throughput and detection accuracy, and have been increasingly adopted for crop-protection monitoring on unmanned aerial vehicles and agricultural edge devices (Rana and Vaidya, 2026; Ahmed et al., 2026; Yu et al., 2025). However, when these models are transferred from controlled laboratory settings to open-field conditions, detection performance often degrades noticeably. The root cause is not merely a data-distribution shift; it lies in the limited ability of general-purpose vision architectures to adapt their representations to the physical properties of field-level disease targets.

Cotton pathology studies show that pathogen spread within leaf tissue is strongly constrained by vascular-bundle architecture. Verticillium dahliae and similar pathogens migrate preferentially along xylem vessels in the longitudinal direction, while lateral diffusion is physically blocked by bundle-sheath walls (Cheng et al., 2025; Zhang et al., 2025b; Li et al., 2025). This constraint produces macroscopically anisotropic lesion contours aligned with primary veins, a pattern documented across multiple host plants (Zhang et al., 2025b; Li et al., 2025). The CCLD dataset constructed in this study corroborates this pattern quantitatively: 67.5% of bounding-box annotations have aspect ratios exceeding 3:1. Principal-axis orientations are approximately uniformly distributed across horizontal, vertical, and diagonal bands, confirming that anisotropic propagation is multi-directional (Cheng et al., 2025; Zhang et al., 2025b). These physical properties impose distinct adaptation requirements on three stages of the detection pipeline: feature extraction, cross-layer transfer, and coordinate regression.

In feature extraction, the YOLO family has undergone successive improvements in inference efficiency (Wang et al., 2024b, Wang et al., 2024a; Khanam and Hussain, 2024; Tian et al., 2025). Lightweight approaches tailored to agricultural disease detection — including strategies for multi-species generalization (Miao et al., 2025) — have also advanced through structural re-parameterization (Ding et al., 2021) and ghost feature generation (Han et al., 2020). However, feature aggregation in all these methods relies on isotropic square kernels, which lack the directional coverage needed for elongated lesions that propagate along vascular bundles. Deformable convolution (DCN) (Zhu et al., 2019) offers a general solution to receptive-field mismatch by learning spatial offsets for adaptive sampling. However, its irregular memory-access patterns cause a substantial drop in inference efficiency under TensorRT (Xiong et al., 2024b). On moderately sized datasets, the offsets also tend to converge poorly, causing the operation to collapse into a standard convolution. Beyond geometric mismatch, open-field confounders such as dead-leaf debris and soil splashes closely resemble early-stage micro-lesions in visual appearance (Zhang et al., 2022b). This similarity highlights the need to separate weak high-frequency pathological signals from the low-frequency background in the spectral domain. Existing approaches either rely on global frequency-domain transforms (e.g., FcaNet (Qin et al., 2021)) at considerable computational cost or adopt fixed frequency groupings (e.g., Octave Convolution (Chen et al., 2019)) that cannot adapt to specific pathological signals. Neither strategy is suitable for lightweight deployment on edge devices.

Beyond these feature-domain mismatches, the irreversible loss of spatial information during multi-stage downsampling poses another critical bottleneck. Early-stage cotton micro-lesions typically occupy only 8–15 pixels at the standard 640×640 input resolution; after successive reduction through the backbone, their local activations nearly vanish in deep feature maps. SPD-Conv (Sunkara and Luo, 2022) avoids the direct spatial discarding of strided convolutions through space-to-depth (S2D) folding, while Gold-YOLO (Wang et al., 2023) and BiFPN (Tan et al., 2020) construct paths for cross-layer information transfer at multiple scales. However, SPD-Conv applies uniform aggregation to all channels after folding, so the few channels carrying high-frequency pathological information are easily diluted by the dominant low-frequency background channels. At the same time, the upsampling path in feature pyramids is inherently an interpolation operation and cannot reconstruct boundary details already lost during forward propagation. In related fields, explicit boundary modeling (Qiu et al., 2020; Yuan et al., 2020) and fine-grained boundary prediction (Kirillov et al., 2020) have been shown to improve localization accuracy. However, the computational paradigms they require—pixel-level label replacement or point-wise rendering—conflict with the throughput demands of real-time detection.

Even when feature extraction and cross-layer fusion are improved, the coordinate regression stage itself retains a geometric adaptation deficiency. Under heterogeneous micro-environmental conditions in the field, lesion contours frequently exhibit pronounced unilateral offset or local distortion. Rotated bounding box methods (Xie et al., 2021) can enclose directional targets more compactly, but rotated NMS introduces 40%–60% additional latency on edge platforms such as Jetson (Yang et al., 2021); we verify this HBB-versus-OBB trade-off empirically in **Table S14**. Moreover, cotton lesions lack a stable principal-direction definition, making annotation consistency difficult to guarantee. We therefore tackle anisotropic adaptation within the horizontal bounding box (HBB) framework by refining the loss function. Current mainstream IoU-family losses (from CIoU (Zheng et al., 2020) and EIoU (Zhang et al., 2022a) to Wise-IoU (Xiong et al., 2024a) and Shape-IoU (Zhang et al., 2025a)) have progressively increased the decoupling granularity of their penalty terms, moving from joint width–height penalization to independent two-dimensional width and height decomposition. Yet the underlying mathematical framework still rests on an implicit isotropic assumption. EIoU, for example, penalizes width and height independently but still treats each as a single regression unit. It cannot distinguish whether a width error originates from the left or the right boundary. When a lesion undergoes extreme offset along a single direction due to physical blockage by a leaf vein, the gradient signal is symmetrically distributed to both boundaries, limiting correction efficiency.

Taken together, existing detection methods lack adaptation to the anisotropic propagation characteristics of field disease targets across three stages: receptive-field geometry, cross-layer information transfer, and boundary regression. To address this gap, we propose a cotton leaf disease detection framework designed for irregular lesion localization under field conditions. The framework improves detection at the feature-extraction, cross-layer transfer, and coordinate-optimization stages through lightweight feature adaptation, multi-scale detail preservation, and boundary-decoupled regression.

The central insight of this work is that high-frequency boundary information constitutes a unified thread linking all three stages of the detection pipeline. In feature extraction, high-frequency lesion-edge signals must be separated from the low-frequency background (AMCA). Before these signals are lost to downsampling, they must be explicitly captured and transferred across layers (DSBT). During multi-scale fusion, the transferred signals must survive channel compression without being diluted (MFS-FPN). At the regression endpoint, the surviving boundary information must be mapped to physically meaningful coordinate corrections rather than coupled geometric abstractions (ABD-IoU). These four modules form a synergistic pipeline in which each stage builds upon the representations established by the preceding one. Ablation experiments confirm super-additive interactions between module pairs, indicating functional complementarity rather than independent contributions.

Although the proposed AMCA, DSBT, MFS-FPN, and ABD-IoU draw on ideas from re-parameterization, ghost feature generation, FPN-style fusion, and IoU-family regression, each module differs from prior art in its core mechanism. HFC-Ghost replaces redundant-feature generation with high-frequency residual extraction; DSBT introduces a dedicated boundary-prior pyramid that bypasses interpolation-based recovery; MFS-FPN performs frequency-aware reduction entirely in the spatial domain without FFT; and ABD-IoU decomposes regression onto four independent physical boundaries in (L, R, T, B) space rather than the conventional (W, H) coupling. Their interactions are functionally non-redundant: ablation experiments (Section 3.2) reveal super-additive synergy in two module pairs, indicating that the gains do not arise from straightforward stacking.

The main contributions are as follows:

- At the feature-extraction stage, we develop a lightweight backbone module (AMCA) for anisotropic feature adaptation. AMCA combines re-parameterizable strip convolutions with high-frequency residual enhancement to strengthen direction-aware lesion representation and local contrast while remaining deployment-friendly after structural folding.
- At the transfer and fusion stages, we design feature-refinement strategies that preserve boundary and detail information during multi-scale transmission. DSBT captures shallow boundary priors before severe downsampling and injects them into the neck, while MFS-FPN refines multi-scale features through spatial-domain operations compatible with edge hardware, helping preserve lesion details under complex field backgrounds.
- At the regression stage, we propose an Anisotropic Boundary-Decoupled IoU loss (ABD-IoU) for irregular lesion localization. Unlike conventional IoU-family losses that penalize box mismatch through coupled geometric terms, ABD-IoU introduces independent penalties on the four physical boundaries of the predicted box. It also applies a logarithmic sustained-modulation factor to improve boundary refinement in high-IoU regimes.
- We evaluate the framework extensively on one self-constructed and two public datasets, together with edge-deployment experiments. Results on CCLD, PlantDoc, and RWD show consistent improvements over strong real-time baselines, and TensorRT deployment on Jetson platforms verifies the practical efficiency of the proposed framework. Furthermore, to facilitate future research, our source code, trained models, and implementation details have been made publicly available at https://github.com/DynaVLA/ABAD-CLD.

## 2 Materials and methods

### 2.1 Overall architecture

**Figure 1:**
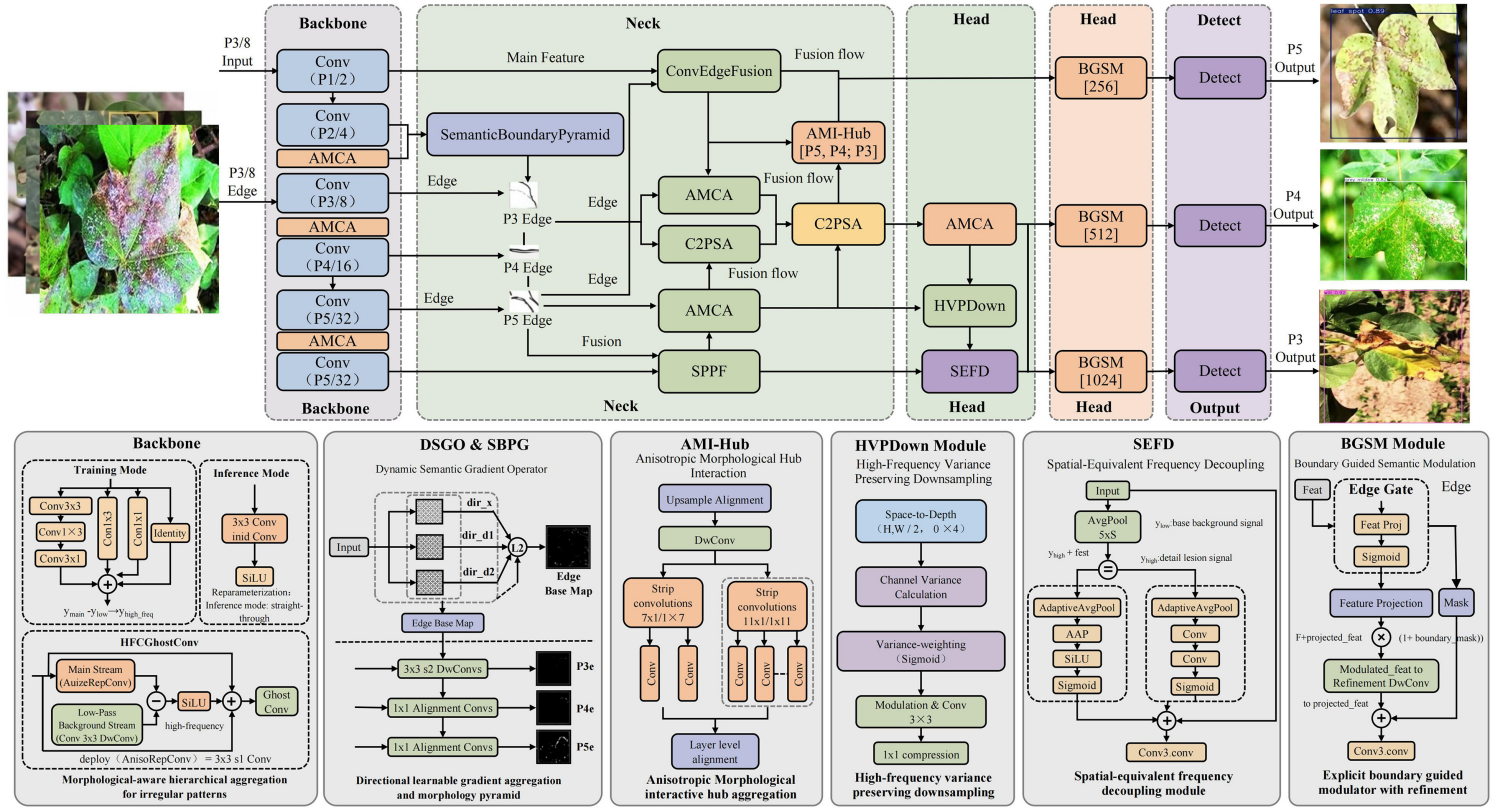
Overall architecture of the proposed anisotropic boundary-aware detection framework.

To address these problems, we propose an anisotropic boundary-aware detection framework based on joint morphological–spectral feature learning. The framework comprises four synergistic modules embedded at different stages of the detection pipeline (**Figure 1**): (1) the AMCA module is deployed at multiple stages of the backbone to establish anisotropic receptive fields and apply high-frequency contrast enhancement at the feature-extraction front end; (2) the DSBT mechanism extracts boundary priors from the shallow backbone output and injects them across layers into the corresponding scales of the neck; (3) MFS-FPN, constructed in the neck, performs frequency-aware downsampling at the channel level and multi-scale morphological feature interaction; (4) the prediction head adopts ABD-IoU loss to perform anisotropic boundary regression for irregular targets.

These four modules address distinct bottlenecks at successive stages of the detection pipeline: AMCA handles front-end representation adaptation; DSBT preserves boundary priors before severe downsampling and transfers them across layers; MFS-FPN maintains detail and frequency selectivity during multi-scale fusion; and ABD-IoU decomposes the final regression error into four individual physical boundaries. Each module consumes the output of its predecessor in a directed chain: AMCA produces high-frequency features → DSBT captures their boundary positions → MFS-FPN preserves them through channel compression → ABD-IoU maps them to per-boundary corrections. Section 3.2 provides empirical evidence that removing any link degrades performance non-additively, supporting the functional non-redundancy of this design.

### 2.2 Anisotropic morphological contrast aggregation module

**Figure 2:**
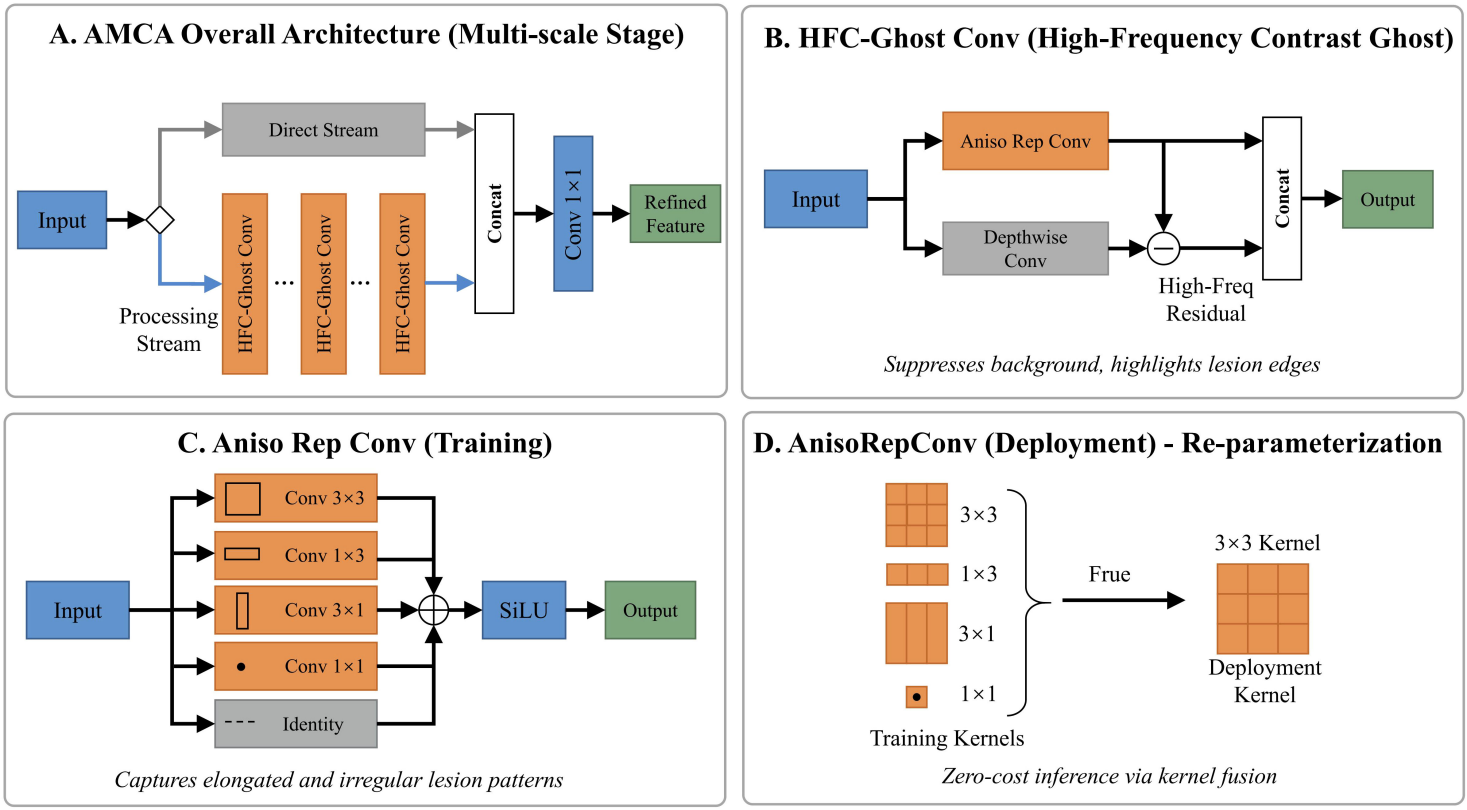
Architecture of the anisotropic morphological contrast aggregation module. **(A)** Overall multi-scale architecture of AMCA based on the split–concat aggregation strategy. **(B)** The HFC-Ghost branch, which extracts a high-frequency residual to suppress background and emphasise lesion edges. **(C)** The training-time anisotropic re-parameterisation branch, in which the 3×3, 1×3, 3×1 and 1×1 convolutions with an identity connection capture elongated and irregular lesion patterns. **(D)** The deployment-time equivalent kernel, where the multi-branch training kernels are folded into a single 3×3 kernel for zero-cost inference.

In standard lightweight networks, tiny lesion activations are easily diluted by the visually homogeneous background of healthy green foliage in field images. The AMCA module addresses this in two ways: it improves feature extraction with directional sensitivity through anisotropic re-parameterization, and it enhances local lesion–background contrast through a high-frequency residual branch. Both operations add only minimal extra overhead at inference time. **Figure 2** shows the overall architecture of AMCA. Subfigure (A) depicts the main path for multi-scale feature aggregation based on the Split-Concat strategy. Subfigures (C) and (D) detail how the training-time asymmetric convolution branches are constructed and equivalently folded into a single 3×3 kernel at inference time. Subfigure (B) illustrates the high-frequency residual extraction mechanism of the HFC-Ghost branch.

#### 2.2.1 Anisotropic structural re-parameterization

Structural re-parameterization, introduced systematically in RepVGG (Ding et al., 2021) and extended to asymmetric branches (1×3, 3×1) by ACNet (Ding et al., 2019), provides the foundation for our approach. We adapt this paradigm to the morphological observation that cotton lesions propagate anisotropically along leaf veins. At training time, we construct a set of multi-branch morphological convolution operators and co-design them with the HFC-Ghost module to jointly optimize morphological adaptation and high-frequency enhancement. The operator group comprises a central 3×3 standard convolution, a horizontal 1×3 strip convolution, a vertical 3×1 strip convolution, and a 1×1 pointwise convolution. All branches share the same input, and each is equipped with an independent batch normalization (BN) layer. At inference time, because convolution and BN are both linear operations, all branch weights are losslessly folded into an equivalent single 3×3 kernel via zero-padding alignment.

Formally, let X denote the input feature map, $W_{k}$ the convolution weight of branch k, and $\mu_{k}$, $\sigma_{k}$, $\gamma_{k}$, $\beta_{k}$ the mean, variance, scaling factor, and bias of the corresponding BN layer. After absorbing the BN parameters, the equivalent convolution weight ${W^{\prime }}_{k}$ and bias ${b^{\prime }}_{k}$ are computed as in Equation 1:

(1) $${W^{\prime }}_{k}=\frac{\gamma_{k}}{\sigma_{k}}\cdot W_{k},\;{b^{\prime }}_{k}=\gamma_{k}\cdot \frac{b_{k}-\mu_{k}}{\sigma_{k}}+\beta_{k}$$

Let the branch set be *K* = {3 × 3,1 × 3,3 × 1,1 × 1}. The final equivalent weight *W_eq_* and bias *b_eq_* after fusion are given by Equation 2:

(2) $$W_{eq}=\sum_{k\in K}P({W^{\prime }}_{k})+{W^{\prime }}_{id},\;b_{eq}=\sum_{k\in K}{b^{\prime }}_{k}+{b^{\prime }}_{id}$$

where *P*(·) is the zero-padding operator that spatially aligns each asymmetric kernel to the 3 × 3 center, and ${W^{\prime }}_{id}$ is the fused parameter of the identity mapping branch.

#### 2.2.2 High-frequency contrast ghost module

In the original GhostNet (Han et al., 2020) design, the auxiliary branch generates redundant feature maps through cheap linear transformations to reduce computation. We redesign this branch as a High-Frequency Contrast Ghost (HFC-Ghost) module that extracts high-frequency residuals instead of producing redundant copies.

Specifically, let *F_main_* denote the morphological feature tensor output by the main branch. The auxiliary branch first performs local spatial aggregation on *F_main_* through a 5 × 5 depthwise separable convolution (DWConv). This kernel is initialized with a uniform distribution, making it functionally approximate a local mean filter at the start of training; it is then adaptively adjusted end-to-end via backpropagation. Two pieces of evidence support the validity of this approximate low-pass assumption. First, uniform initialization provides a low-pass-biased starting point. Second, after training convergence, the 2D-FFT magnitude spectrum of the learned kernel shows energy concentrated in the low-frequency central region (**Figure S2**; **Table S10**).

The residual tensor *F_high_* is then extracted by element-wise subtraction of this low-frequency component from *F_main_*. Because subtraction shifts the output distribution toward large negative values, the subsequent SiLU activation—whose response diminishes exponentially in the negative regime—would suppress most of the high-frequency residual signal. A BN layer is therefore inserted after the subtraction to re-center the distribution around zero, which normalizes the output to a zero-mean, unit-variance distribution and ensures that the subsequent SiLU (Sigmoid Linear Unit) activation operates over its full effective range, as in Equation 3:

(3) $$F_{high}=BN(F_{main}-DWConv_{5\times 5}(F_{main}))$$

This design suppresses low-frequency background interference early in feature generation while strengthening high-frequency responses associated with lesion edges. The goal is to extract high-frequency residuals and concatenate them with the main-branch features to enrich the representation; this differs from the gain operation in unsharp masking (USM). FcaNet (Qin et al., 2021) applies channel attention based on DCT basis functions, and Octave Convolution processes multi-resolution features through fixed high/low-frequency grouping. In contrast, HFC-Ghost extracts high-frequency residuals directly at the original resolution via learnable spatial subtraction. It requires neither frequency-domain transforms nor fixed grouping, making it better suited for edge deployment.

### 2.3 Dynamic semantic boundary transfer

**Figure 3:**
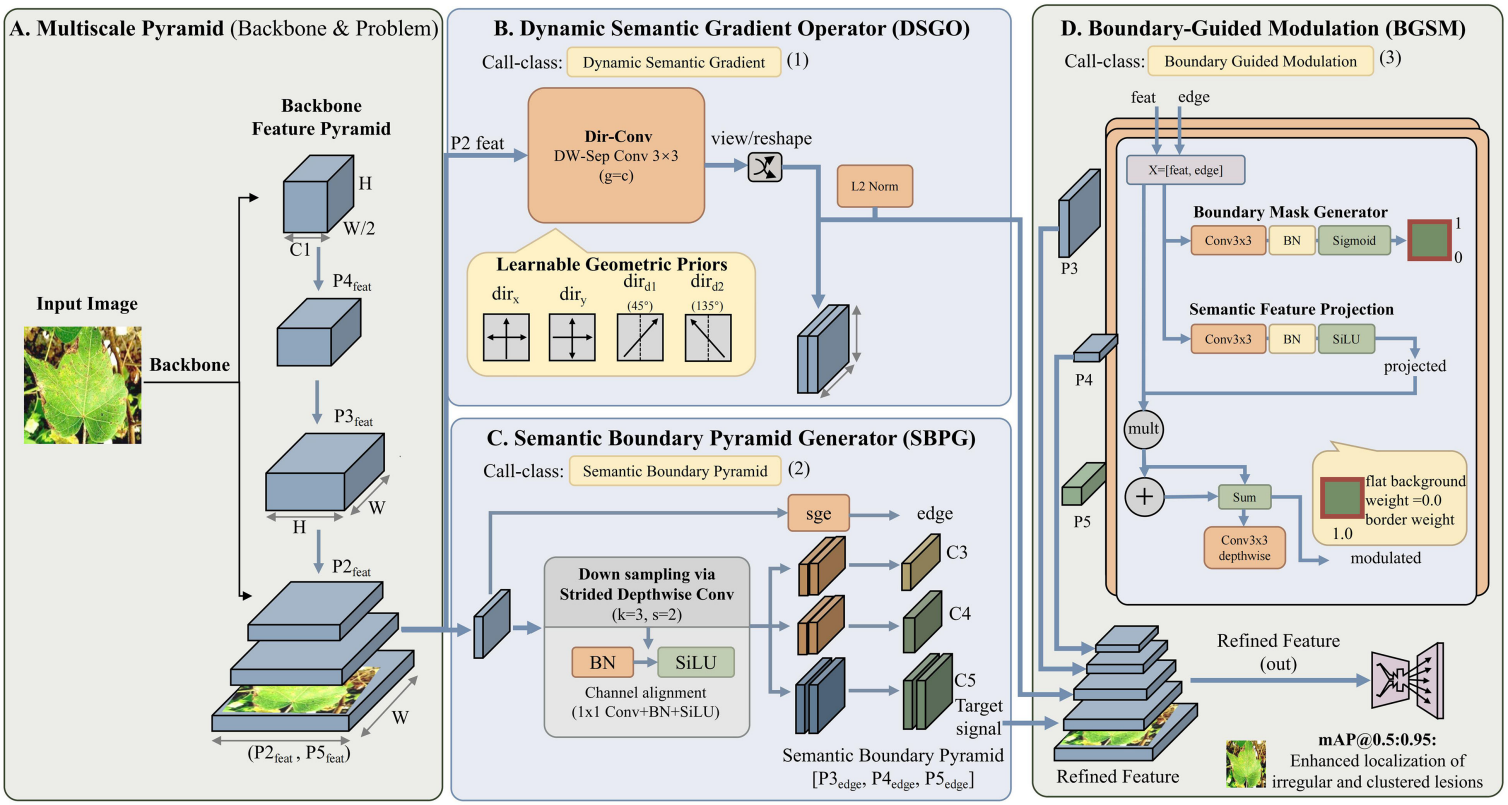
Structure of the dynamic semantic boundary transfer mechanism. **(A)** The multiscale backbone feature pyramid providing the input features. **(B)** The dynamic semantic gradient operator (DSGO), which computes multi-directional gradients using learnable geometric priors. **(C)** The semantic boundary pyramid generator (SBPG), which produces a multi-scale boundary-prior pyramid through strided depthwise downsampling and channel alignment. **(D)** Boundary-guided modulation (BGSM), which converts the boundary priors into a spatial mask that refines the deep features for the P3–P5 levels.

The AMCA module enhances local contrast in lesion regions but does not explicitly encode boundary positions, and its effect is confined to a single feature layer. As multi-level downsampling proceeds, fine-grained boundary information undergoes progressive, irreversible attenuation that interpolation-based upsampling in conventional FPNs cannot recover. To address this, we propose the DSBT mechanism. DSBT extracts boundary priors from shallow feature maps using learnable gradient operators and transfers them independently to each scale level through a dedicated progressive downsampling pyramid. These priors are then injected into deep semantic features via spatial multiplicative modulation. Unlike a conventional FPN, which relies primarily on upsampling to recover shallow-layer details, DSBT captures boundary priors before severe downsampling and delivers them to the corresponding scale through a dedicated branch. **Figure 3** illustrates the overall design. Subfigure (B) details semantic gradient extraction via multi-directional grouped convolution. Subfigure (C) shows how the SBPG module generates a multi-scale boundary prior pyramid through stride-based progressive downsampling. Subfigure (D) shows how the BGSM module converts boundary priors into a spatial modulation mask that softly enhances deep features.

#### 2.3.1 Multi-directional semantic gradient operator

We construct a set of learnable gradient operators in four directions, initializing the differential weights at polar angles $\text{Θ}=\{0^{\circ },90^{\circ },45^{\circ },135^{\circ }\}$. Four directions strike a balance between directional coverage and parameter efficiency, as they already capture the principal spatial gradient orientations. Increasing the count to six or eight yields only marginal accuracy gains while proportionally increasing both parameter count and computational cost (**Table S9**).

The proposed gradient operators differ from classical Sobel/Canny operators in two respects. First, their weights are initialized from directional differential priors but then optimized via backpropagation, enabling them to learn more discriminative boundary representations in the semantic feature space. Second, the operators are applied depthwise, preserving channel independence with a minimal parameter count.

The DSGO is implemented as a depthwise 3 × 3 convolution with groups equal to the channel count *C*, stride 1, padding 1, and no bias. The four directional kernels are initialized from analytical first-order differential templates aligned with $\text{Θ}=\{0^{\circ },90^{\circ },45^{\circ },135^{\circ }\}$ and are then updated by standard backpropagation with the same base learning rate as the rest of the model. The stability constant *ϵ* in Equation 4 is set to *ϵ* = 1×10^−6^, matching standard FP32 numerical-stability practice for the square-root aggregation. This configuration introduces 6.4K learnable parameters in total—approximately 0.24% of the YOLOv11n backbone parameter count—and 0.01GFLOPs at the 640 × 640 input resolution.

Given the input semantic feature tensor $F_{sem}\in {\mathbb{R}}^{C\times H\times W}$, the aggregated boundary magnitude *M_edge_* is obtained by combining the directional gradient responses via the L_2_ norm, as in Equation 4:

(4) $$M_{edge}=\sqrt{\sum_{i\in \text{Θ}}{\left(\nabla_{i}*F_{sem}\right)}^{2}+\epsilon }$$

where ∇*_i_* is the depthwise convolution operator for direction *i* and *ϵ* is a stability constant that prevents numerical underflow. The L_2_ norm ensures that opposite-sign gradient responses do not cancel, providing a reliable estimate of semantic boundary magnitude.

The L_2_ norm aggregates directional gradients because DSBT is responsible only for generating a direction-agnostic boundary presence mask; directional adaptation has already been completed by AMCA during feature extraction and is implicitly encoded in the channel activations. Retaining explicit directional information would double the channel count, exceeding the computational budget for edge deployment.

#### 2.3.2 Semantic boundary pyramid generator

The boundary priors from the multi-directional gradient operators reside at shallow-network resolution; adapting them to the P3, P4, and P5 scale levels in the neck requires multi-level downsampling. Because conventional max pooling discards sub-pixel boundary displacement information, the boundary pyramid generator instead performs progressive spatial reduction using depthwise separable convolutions with stride 2 (*k* = 3*,s* = 2). A 1 × 1 convolution followed by BN then aligns the channel dimension.

Let the initial boundary feature be *E*_0_ = *M_edge_*. The boundary prior feature *E_i_* at each scale level *i* ∈ {1,2,3}, corresponding to P3, P4, and P5, is generated by the following recurrence in Equation 5:

(5) $$E_{i}=\sigma (ℬ(Conv_{1\times 1}(\sigma (ℬ(DWConv_{3\times 3,s=2}(E_{i-1}))))))$$

where *ℬ*(·) denotes batch normalization and *σ*(·) the SiLU activation function. The resulting multi-scale boundary prior set {*E*_1_*, E*_2_*, E*_3_} is spatially aligned with each corresponding level of the neck.

#### 2.3.3 Boundary-guided spatial modulation

Conventional feature fusion mixes boundary and semantic information with equal weight, leaving no room for spatially differentiated enhancement. The BGSM module addresses this limitation through multiplicative spatial modulation: the boundary response map is first projected to a 2D spatial mask valued in (0, 1), which then applies position-adaptive gain control to the deep features.

Specifically, the boundary mask Mask_boundary_ is generated by the following transformation in Equation 6:

(6) $${\text{Mask}}_{\text{boundary}}=\sigma_{\text{Sigmoid}}(ℬ({\text{Conv}}_{3\times 3}(F_{\text{edge}})))$$

The target semantic features first undergo channel projection for dimension alignment, then spatial modulation and residual refinement, as in Equation 7:

(7) $$F_{\text{proj}}\;=\;{\text{Conv}}_{1}\times 1(F_{\text{sem}})\;\hat{F}=\;F_{\text{proj}}\;+\;{\text{Conv}}_{\text{refine}}\;F_{\text{proj}}\;⊗\;(1\;+\;{\text{Mask}}_{\text{boundary}})\;$$

Where the boundary response is strong, Mask_boundary_ approaches 1 and the feature value at that location can theoretically receive nearly twofold gain. In practice, the Sigmoid output mostly falls within the 0.3–0.7 range, so the actual gain magnitude is determined adaptively during training; in flat background regions, feature values remain unchanged. The additive residual connection ensures training stability throughout this modulation. The additive constant 1 serves as an identity base gain, so that modulation acts as residual enhancement rather than hard gating: where he gain equals 1 and features pass through unchanged; where ${\text{Mask}}_{\text{boundary}}\to 1$, the gain approaches 2. Ideally, the mask maintains a low response over background regions, concentrating modulation on boundary-related positions.

### 2.4 Morphological–spectral synergistic feature pyramid network

**Figure 4:**
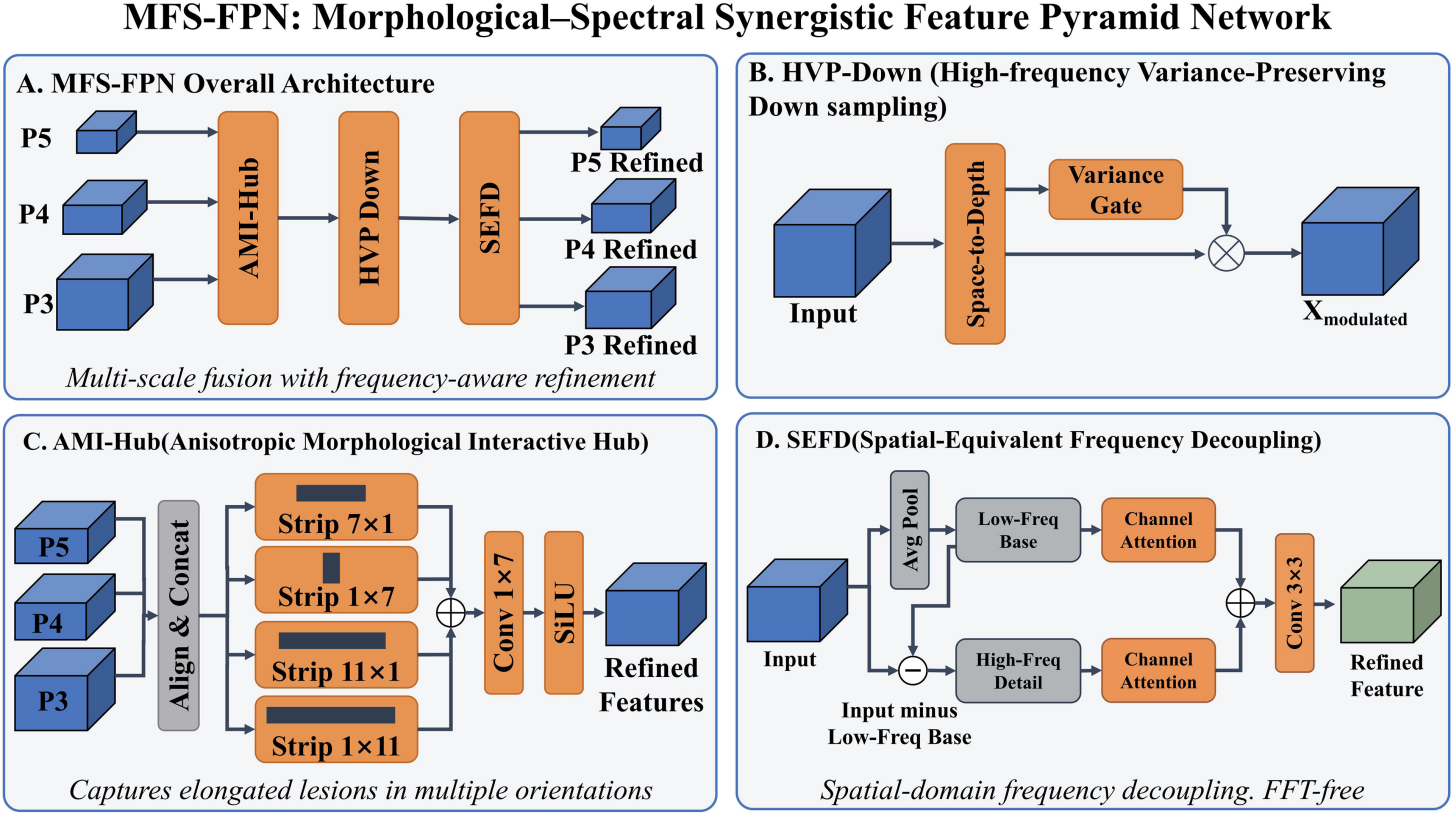
Architecture of the morphological–spectral synergistic feature pyramid network.

The neck must perform multi-scale fusion and separate high- from low-frequency signals under hardware constraints that preclude FFT-based operations. We therefore design MFS-FPN, which comprises three submodules illustrated in **Figure 4**. Multi-scale features first pass through the Anisotropic Morphological Interaction Hub (AMI-Hub; subfigure C) for directional morphological interaction. The High-frequency Variance-Preserving Downsampling module (HVP-Down; subfigure B) then performs frequency-aware channel-gated dimensionality reduction, and the Spatial-Domain Spectral Decoupling (SEFD; subfigure D) strips residual low-frequency interference at the fusion endpoint. The three submodules are cascaded in the order AMI-Hub → HVP-Down → SEFD so that morphological interaction occurs in the full-channel space before channel compression and frequency stripping.

#### 2.4.1 Anisotropic morphological interaction hub

For diseases with radial or elongated propagation patterns, such as wilt, the morphological interaction module replaces the simple feature-alignment concatenation used in conventional necks. Given the fused multi-scale feature *F_fusion_*, the module applies orthogonal asymmetric strip convolutions covering mid-range (*k* = 7) and long-range (*k* = 11) receptive fields to capture directional edge expansion at multiple scales with minimal parameter and computational overhead, as in Equation 8:

(8) $$F_{out}=\sigma_{SiLU}(F_{fusion}+\sum_{k\in \{7,11\}}(Conv_{k\times 1}(F_{fusion})+Conv_{1\times k}(F_{fusion})))$$

The residual connection preserves the integrity of the main fusion pathway, and the SiLU activation introduces nonlinearity to strengthen the representational capacity of directional features.

#### 2.4.2 High-frequency variance-preserving downsampling

Because AMCA suppresses the low-frequency background, the channel-wise spatial variance after AMCA correlates more reliably with the high-frequency pathological response. This variance can therefore serve as an approximate proxy for high-frequency energy (**Table S11**; **Figure S3**). This approximation holds only if that suppression is sufficient. The two modules are naturally coupled through gradient propagation during end-to-end training. On this basis, HVP-Down computes the spatial variance of each channel after space-to-depth (S2D) folding and uses the result, defined in Equation 9, as a proxy for information variation within that channel:

(9) $$Var_{c}(X_{folded})=\frac{1}{H\times W}\sum_{h=1}^{H}\sum_{w=1}^{W}{\left(X_{c,h,w}-\mu_{c}\right)}^{2}$$

where *H* and *W* are the spatial dimensions of the feature map after S2D folding, and *µ_c_* is the pixel mean of channel *c*.

A multi-layer perceptron (MLP) then converts the channel variance vector into gating weights that apply differentiated scaling to each channel, as in Equation 10:

(10) $$X_{out}=Conv(X_{folded}⊙(1+MLP(Var(X_{folded}))))$$

This gating mechanism assigns larger retention weights to channels with higher variance during cross-scale dimensionality reduction, mitigating the signal dilution caused by indiscriminate channel aggregation in standard SPD-Conv.

#### 2.4.3 Spatial-domain spectral decoupling

This module strips residual low-frequency background artifacts such as specular highlights and soil at the output of MFS-FPN. It exploits the low-pass approximation property of mean pooling to separate high- and low-frequency components coarsely in the spatial domain, providing a hardware-friendly frequency-aware alternative that avoids FFT entirely. By the convolution theorem, a *k* × *k* uniform mean pooling operation corresponds to a sinc-type low-pass transfer function in the frequency domain whose first zero lies at spatial frequency *f* = 1*/k*. With *k* = 5, components below approximately 20% of the Nyquist frequency are suppressed, so the extracted *F_base_* approximately represents DC and near-DC spectral content. The frequency selectivity of this single-stage decomposition is lower than that of wavelet or FFT methods. However, it requires only one mean pooling and one subtraction, introduces no learnable parameters, and is well-suited to edge platforms that lack native FFT hardware.

Specifically, a k×k local mean pooling extracts the low-frequency base component $F_{base}$ (*k* = 5) in this work. Element-wise subtraction then yields the high-frequency residual $F_{detail}$ = $F_{in}$− $F_{base}$. Finally, global adaptive pooling generates channel attention weights $A_{high}$and $A_{low}$ for the high- and low-frequency components, which are combined by weighted fusion as in Equation 11:

(11) $$F_{refined}=(A_{high}(F_{detail})⊙F_{detail})+(A_{low}(F_{base})⊙F_{base})$$

### 2.5 Anisotropic boundary-decoupled IoU loss

**Figure 5:**
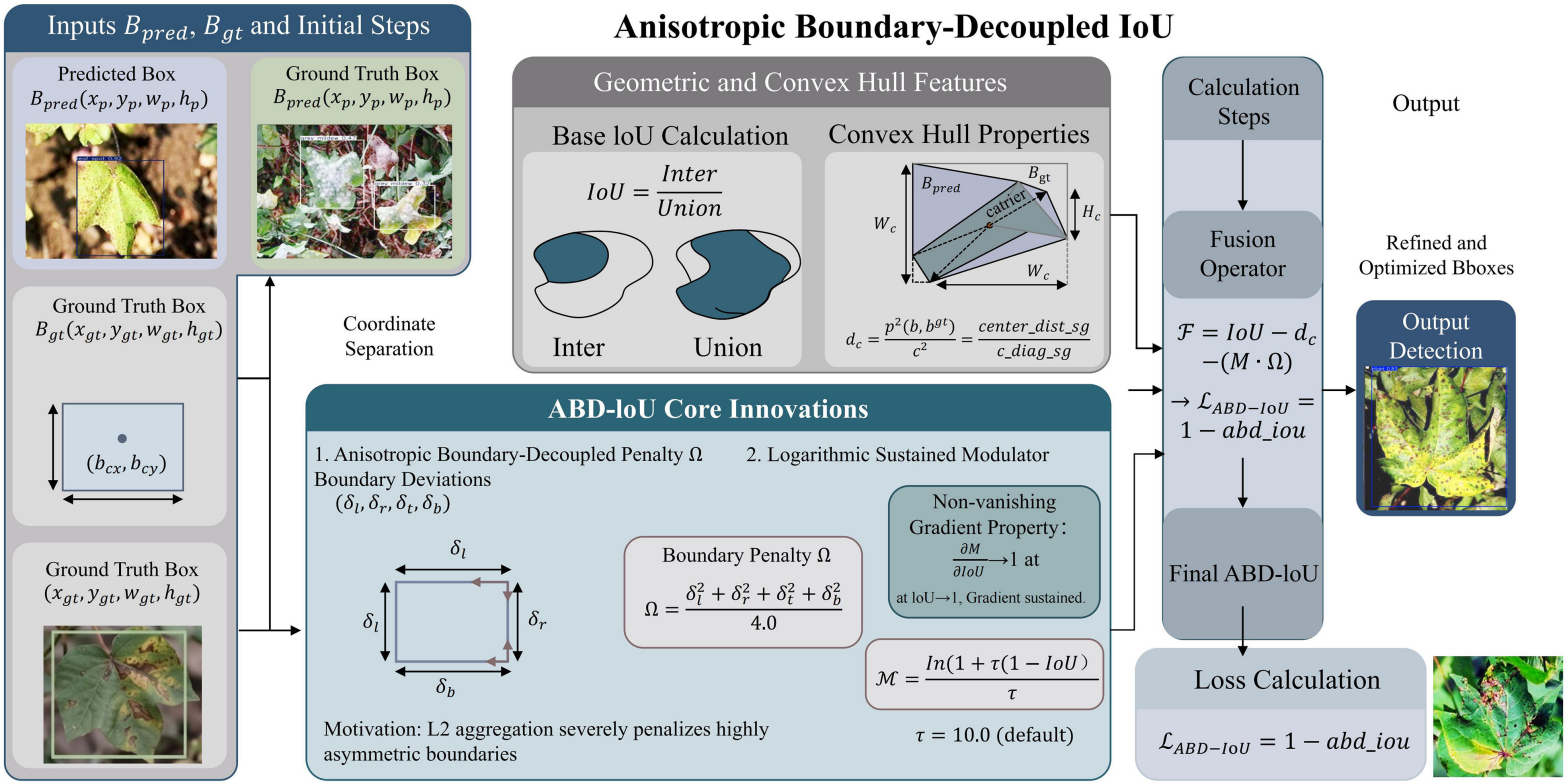
Schematic illustration of the anisotropic boundary-decoupled IoU loss.

MFS-FPN provides the detection head with frequency-refined semantic feature maps. At the regression stage, however, the isotropic penalty structure of conventional IoU-family losses remains a bottleneck for localization accuracy. ABD-IoU (**Figure 5**) addresses this in two respects: it decomposes the regression penalty onto each of the four physical boundaries, and it alleviates gradient vanishing in the high-IoU range.

#### 2.5.1 Four-boundary independent penalty

The detection framework adopts an anchor-free paradigm in which the head outputs the distances from the center point to the four boundaries (*L, R, T, B*). Existing approaches typically convert (*L, R, T, B*) back to (*X, Y, W, H*) coordinates before computing CIoU or EIoU losses; this transformation reintroduces inter-boundary coupling in the penalty terms. ABD-IoU instead constructs penalty terms directly in (*L, R, T, B*) space, avoiding the gradient coupling caused by coordinate conversion. It discards the coupled aspect-ratio penalty and independently computes the relative deviation ∆*_i_* between the predicted and ground-truth boxes on each boundary, constructing the penalty term *P*_bound_ with the L_2_ norm, as in Equation 12:

(12) $$P_{\text{bound}}=14\sum_{i\in \{L,R,T,B\}}{\left(\frac{{\text{Δ}}_{i}}{C_{i}}\right)}^{2}$$

where *C* $C_{i}$ takes the width $C_{w}$ of the minimum enclosing rectangle when i∈{L,R} and its height $C_{h}$ when i∈{T,B}. The squared ${\text{L}}_{2}$ norm imposes a stronger penalty on larger boundary deviations, so the optimization tends to correct the most displaced boundaries first.

#### 2.5.2 Logarithmic sustained modulation factor

IoU-based losses suffer from gradient vanishing when predicted and ground-truth boxes overlap heavily, causing boundary fine-tuning to saturate in later training stages. To address this, a logarithmic sustained modulation factor M (IoU) is introduced, as in Equation 13:

(13) $$M(\text{IoU})=\frac{ln\;(1+\tau (1-IoU))}{\tau }$$

where τ is a temperature parameter controlling the logarithmic curvature, we fix τ=10 in all experiments, selected from the sweep over τ∈{1,5,10,20,50} (**Table S6**); τ=10 gives the strongest performance in the high-IoU regime without compromising training stability. Under this setting, the partial derivative defined in Equation 14 yields a gradient magnitude

(14) $$|\frac{\partial M}{\partial IoU}|=\frac{1}{1+\tau (1-IoU)}$$

which increases monotonically from $\frac{1}{1+\tau }\approx 0.091$ at IoU=0 to 1.0 at IoU=1. This is the central property of LSM: the optimization signal is strongest exactly where standard IoU-based losses lose gradient. Paired with the cosine learning-rate annealing schedule (Section 3.1.1), the residual parameter update η(t)·g still vanishes as η(t)→0, so the sustained-gradient property accelerates fine-grained boundary refinement without inducing late-stage oscillation.

When IoU approaches 1, the gradient magnitude remains a non-zero constant, sustaining an effective optimization signal. *τ* directly controls gradient preservation in the high-IoU range: a larger *τ* causes faster decay in the mid-IoU range but stronger preservation near IoU = 1, whereas a smaller *τ* yields a flatter gradient curve.

A natural concern is whether this non-vanishing limiting gradient might impede convergence. In practice, it does not, for two reasons. First, field-collected annotations inherently contain pixel-level uncertainty, and the residual gradient from LSM resembles the non-zero loss floor of Flooding regularization (Ishida et al., 2020), which has been shown to suppress overfitting to noisy labels. Second, cosine annealing—a necessary companion setting for ABD-IoU—is applied in later training stages, so the actual parameter update *η*(*t*) · g approaches zero as *η*(*t*) → 0. Following the center-distance penalty paradigm of DIoU, define dc as in Equation 15:

(15) $$d_{c}=\frac{\rho^{2}(b,b^{gt})}{c^{2}}$$

where b and $b^{gt}$ denote the center-point coordinates of the predicted and ground-truth boxes respectively, *ρ*(·,·) is the Euclidean distance, and *c* is the diagonal length of the minimum enclosing rectangle. Combining all three penalty terms, the complete definition of ABD-IoU is given by Equation 16:

(16) $$L_{ABD-IoU}=1-IoU+d_{c}+M(IoU)\cdot P_{bound}$$

The three terms play complementary roles: *d_c_* drives global center-point alignment, *P*_bound_ applies directional corrections independently on each boundary, and M (IoU) sustains gradient effectiveness in the high-IoU range. ABD-IoU deliberately omits the aspect-ratio penalty term *αv* from CIoU because the isotropic structure of *αv* — jointly encoding width and height deviations via cosine similarity — contradicts independent four-boundary decoupling.

### 2.6 Datasets and evaluation protocol

To validate both detection performance and cross-domain generalization, a self-constructed dataset and two public benchmarks are used. Together, they span two evaluation dimensions: cross-scene and cross-crop.

#### 2.6.1 CCLD dataset

Current mainstream public plant disease datasets are mostly collected under controlled laboratory conditions, introducing a significant domain gap relative to field operations. To better approximate real-world field detection, this work constructs the Complex Cotton Leaf Disease dataset (CCLD). All images were captured in open cotton fields in Xinjiang and cover six categories: Blight, Curl, Grey mildew, Healthy, Leaf spot, and Wilt. Three plant-protection specialists annotated the images in two rounds: first independently, then through arbitration-based correction, following the minimum bounding rectangle principle. The resulting mean pairwise bounding-box IoU is 0.82 ± 0.07 and Fleiss’ *κ* is 0.78, indicating substantial inter-annotator agreement. The class imbalance ratio is 2.96: 1 (Wilt vs. Grey mildew); training relies on the inherent class-mixing effect of Mosaic augmentation without explicit resampling.

CCLD is designed to encompass three categories of typical field challenges: (1) difficult background scenes affected by direct highlights and homogeneous dead-leaf interference; (2) high-occlusion and target-overlap scenes under dense planting conditions; and (3) scenes containing extremely early-stage micro-lesions occupying only a few pixels (**Figure S1**; **Table 1**).

**Table 1:** Statistical summary of the CCLD dataset, including class distribution, annotation consistency, and bounding box geometric properties.

Category	Statistic	Value
*General*	Total images	6,856
	Total bounding box annotations	12,428
	Number of categories	6
	Train/Val/Test split ratio	8:01:01
	Native image resolution	3,024×4,032 (smartphone)/4,000×3,000 (UAV)
*Per-class annotation counts*	Blight	2,147
	Curl	1,683
	Grey mildew	1,052
	Healthy	1,896
	Leaf spot	2,534
	Wilt	3,116
Class imbalance	Max/Min class annotation ratio	2.96: 1
*Geometric properties*	Proportion of boxes with aspect ratio ¿ 3:1	67.50%
	Bounding box aspect ratio range (5th–95th percentile)	1.4: 1 – 6.2: 1
	Fraction with principal axis near horizontal (*θ*≤15^◦^)	28.40%
	Fraction with principal axis near vertical (*θ*≥75^◦^)	34.10%
	Fraction with principal axis in diagonal band (15^◦^ *<θ<*75^◦^)	37.50%

Aspect ratio is computed as max (*w,h*)*/*min (*w,h*) for each bounding box. The principal axis orientation *θ* is defined as the angle between the longer side of the minimum bounding rectangle and the horizontal axis, where *θ* = 0^◦^ denotes a horizontally elongated box and *θ* = 90^◦^ denotes a vertically elongated box. The diagonal band (15^◦^ *< θ <* 75^◦^) reflects disease lesions spreading along oblique secondary veins rather than the primary vein axis.

#### 2.6.2 PlantDoc dataset for cross-scene evaluation

To verify cross-species generalization, the PlantDoc dataset (Singh et al., 2020) is introduced for cross-scene evaluation. PlantDoc encompasses 27 disease and healthy-leaf categories across 13 plant species, comprising 2,598 images and 8,595 annotated bounding boxes. Its images are collected from the Internet, presenting a significant domain gap from CCLD in background conditions. Leaf morphologies span a diverse range from dicotyledons to monocotyledons, requiring the model to learn pathological feature representations that generalize across species. Because the two datasets differ in category-definition granularity, cross-domain evaluation is conducted on semantically alignable superclasses. Semantic mapping identifies three such superclasses: Blight, Leaf spot, and Healthy. Although diseases of the same name in different plants may differ in pathogen species, their macroscopic visual phenotypes—tissue necrosis and browning—exhibit cross-species commonality. The cross-domain evaluation examines whether the model can capture this shared representation. Curl, Grey mildew, and Wilt in CCLD have no alignable counterparts in PlantDoc and are therefore excluded.

#### 2.6.3 RWD dataset for cross-crop evaluation

While PlantDoc provides multi-species mixed validation across 13 plants, a stricter generalization constraint is imposed by introducing the Roboflow Wheat Disease object detection dataset (RWD) (Roboflow, 2024), which tests the transferability of the proposed framework on a single unseen crop. RWD contains 1,600 field wheat images with bounding-box annotations covering five categories: Yellow Rust, Brown Rust, Stem Rust, Smut, and Healthy, in a format directly compatible with mainstream detection frameworks. Wheat leaves are narrow, elongated monocotyledon structures with parallel venation, fundamentally different from the broad, palmately lobed leaves and reticulate venation of cotton. The vascular-bundle constraints governing lesion expansion also differ, making this dataset well-suited to examining whether the anisotropic receptive field of AMCA and the boundary gradient extraction of DSBT remain applicable to non-cotton crops.

## 3 Results and analysis

### 3.1 Experimental setup

#### 3.1.1 Implementation details

All experiments were conducted on Ubuntu 22.04 LTS with PyTorch 2.1.0, using a single NVIDIA T4 GPU (16 GB VRAM) for training, validation, and inference. To ensure fair comparison and reproducibility, each compared model was initialized from its own officially released pre-trained weights at the equivalent scale class to avoid penalizing the baselines, and the global random seed was fixed at 42 to eliminate variance from stochastic fluctuations; a statistical-significance test over 10 independent runs against five comparison models is reported in **Table S12**.

All models were trained for 300 epochs with input images resized to 640×640 pixels and a batch size of 32. Mosaic augmentation was disabled during the last 10 epochs to prevent distribution shift from prolonged augmentation in late training. The optimizer was SGD with a momentum factor of 0.937, an initial learning rate of 0.01, weight decay of 5×10^−4^, and a cosine annealing schedule. Because cosine annealing is a required companion setting for the LSM factor in ABD-IoU, all compared methods shared the same schedule to ensure fairness (**Table S13**; **Figure S4**). Unless otherwise stated, all results reported in the main text correspond to the run with the fixed global seed (42). For fair benchmarking, every model in **Table 2**, **Table 3**, and **Table 4** — including general-purpose YOLO baselines and domain-specific variants — was trained under identical hyper-parameters (300 epochs, batch 32, 640×640 input, SGD with momentum 0.937, initial LR 0.01, weight decay 5 × 10^−4^, cosine annealing), the same fixed seed (42), identical train/val/test splits, and identical augmentation policies (Mosaic disabled for the last 10 epochs). Reported FPS values were measured on the same NVIDIA T4 GPU under FP32 precision unless otherwise stated.

**Table 2:** Comparison with existing general-purpose and domain-specific detectors on CCLD, PlantDoc, and RWD.

Model	Year	Type	GFlops	Params (M)	mAP @50	mAP @50:95	AP_S_	∆ mAP@50	FPS
CCLD									
YOLOv8n (Jocher et al., 2023)	2023	General	8.10	3.01	71.20	59.80	26.10	-2.50	228
YOLOv9t (Wang et al., 2024b)	2024	General	7.70	2.00	72.40	60.50	27.30	-1.30	220
YOLOv10n (Wang et al., 2024a)	2024	General	6.70	2.30	72.80	61.20	27.80	-0.90	236
YOLOv11n (Khanam and Hussain, 2024)	2024	General	6.30	2.60	73.70	62.30	28.40	—	218
YOLOv12n (Tian et al., 2025)	2025	General	6.50	2.60	74.10	62.50	28.80	+0.40	212
YOLOv13n (Lei et al., 2025)	2025	General	6.40	2.60	73.91	62.45	28.65	+0.21	208
YOLOv5s (Jocher, 2022)	2021	General	16.00	7.20	74.50	62.10	27.50	+0.80	165
YOLOv11s (Khanam and Hussain, 2024)	2024	General	21.40	9.40	76.30	63.80	30.20	+2.60	142
DEMM-YOLO (Gao et al., 2025)	2025	Cotton	7.80	3.20	75.60	63.10	29.50	+1.90	185
CFNet-YOLOv8s (Li et al., 2024)	2024	Cotton	23.30	11.10	76.80	63.50	29.80	+3.10	98
ACURS-YOLO (Hu et al., 2025a)	2025	Cotton	8.50	3.40	75.90	63.30	30.10	+2.20	178
CM-YOLO (Hu et al., 2025b)	2025	Cotton	9.20	3.80	76.20	63.60	30.60	+2.50	170
LCDDN-YOLO (Feng et al., 2025)	2025	General	7.10	2.80	74.80	62.70	28.90	+1.10	195
**Ours**	**—**	**Improved**	**6.36**	**2.73**	**78.50**	**65.00**	**35.10**	**+4.80**	**202**
PlantDoc									
YOLOv8n (Jocher et al., 2023)	2023	General	8.10	3.01	53.20	33.80	—	-3.10	228
YOLOv9t (Wang et al., 2024b)	2024	General	7.70	2.00	55.10	35.20	—	-1.20	220
YOLOv10n (Wang et al., 2024a)	2024	General	6.70	2.30	54.80	34.90	—	-1.50	236
YOLOv11n (Khanam and Hussain, 2024)	2024	General	6.30	2.60	56.30	35.70	—	—	218
YOLOv12n (Tian et al., 2025)	2025	General	6.50	2.60	56.80	36.00	—	+0.50	212
YOLOv13n (Lei et al., 2025)	2025	General	6.40	2.60	56.75	35.80	—	+0.45	208
YOLOv11s (Khanam and Hussain, 2024)	2024	General	21.40	9.40	58.50	37.40	—	+2.20	142
SerpensGate-YOLOv8 (Miao et al., 2025)	2024	PlantDoc	9.80	4.20	57.90	36.80	—	+1.60	155
YOLOv9++ (Gautam and Prasad, 2024)	2025	PlantDoc	10.50	4.80	58.70	37.10	—	+2.40	148
**Ours**	**—**	**Improved**	**6.36**	**2.73**	**60.80**	**38.90**	**—**	**+4.50**	**202**
RWD									
YOLOv8n (Jocher et al., 2023)	2023	General	8.10	3.01	68.30	42.10	—	-2.80	228
YOLOv9t (Wang et al., 2024b)	2024	General	7.70	2.00	69.50	43.20	—	-1.60	220
YOLOv10n (Wang et al., 2024a)	2024	General	6.70	2.30	69.80	43.50	—	-1.30	236
YOLOv11n (Khanam and Hussain, 2024)	2024	General	6.30	2.60	71.10	44.60	—	—	218
YOLOv12n (Tian et al., 2025)	2025	General	6.50	2.60	71.80	45.00	—	+0.70	212
YOLOv13n (Lei et al., 2025)	2025	General	6.40	2.60	71.50	44.80	—	+0.40	208
YOLOv11s (Khanam and Hussain, 2024)	2024	General	21.40	9.40	74.60	47.30	—	+3.50	142
**Ours**	**—**	**Improved**	**6.36**	**2.73**	**76.80**	**49.50**	**—**	**+5.70**	**202**

Bold values indicate the best result in each column.

**Table 3:** PlantDoc cross-domain direct transfer (no fine-tuning, per-superclass AP@50).

Method	Training data	Test data	Blight AP@50	Leaf Spot AP@50	Healthy AP@50	Mean mAP@50
YOLOv11n	CCLD	PlantDoc	28.50	26.80	38.30	31.20
YOLOv12n	CCLD	PlantDoc	29.20	27.40	39.10	31.90
CM-YOLO	CCLD	PlantDoc	27.80	26.10	37.50	30.47
Ours	CCLD	PlantDoc	33.40	31.60	42.40	35.80
∆	—	—	+4.90	+4.80	+4.10	+4.60

**Table 4:** Zero-shot cross-crop transfer from CCLD to the RWD wheat disease dataset.

Method	Params (M)	FLOPs (G)	mAP@50	mAP@50:95	AP_S_	FPS
YOLOv8n	3.01	8.10	29.80	17.10	7.80	228
YOLOv11n (Baseline)	2.60	6.30	30.90	17.70	8.30	218
YOLOv11n + SPD-Conv	2.80	6.50	32.40	18.90	9.80	208
**Ours (Full Model)**	**2.73**	**6.36**	**38.60**	**22.60**	**14.20**	**202**

Bold values indicate the best result in each column.

#### 3.1.2 Evaluation metrics

The evaluation follows the standard COCO protocol across three complementary dimensions. (1) Accuracy. The core metrics are precision (P), the true-positive fraction among predictions, and recall (R), the fraction of ground-truth positives detected. mAP@50 quantifies overall localization and classification accuracy under complex field backgrounds, while the stricter mAP@50:95 measures how well the boundary-decoupling mechanism localizes irregular lesions. (2) Fine-grained ablation. For the micro-level ablation of ABD-IoU, we also report small-object APS, medium-object APM, and large-object APL to isolate irregular-boundary regression gains at each target scale. (3) Lightweight design and deployment. Because agricultural UAV edge-computing platforms impose hard real-time constraints, we report total parameter count (Params/M), computational complexity (GFLOPs), and inference throughput at batch size 1 to gauge deployment feasibility.

### 3.2 Ablation studies

#### 3.2.1 Progressive module ablation

**Figure 6:**
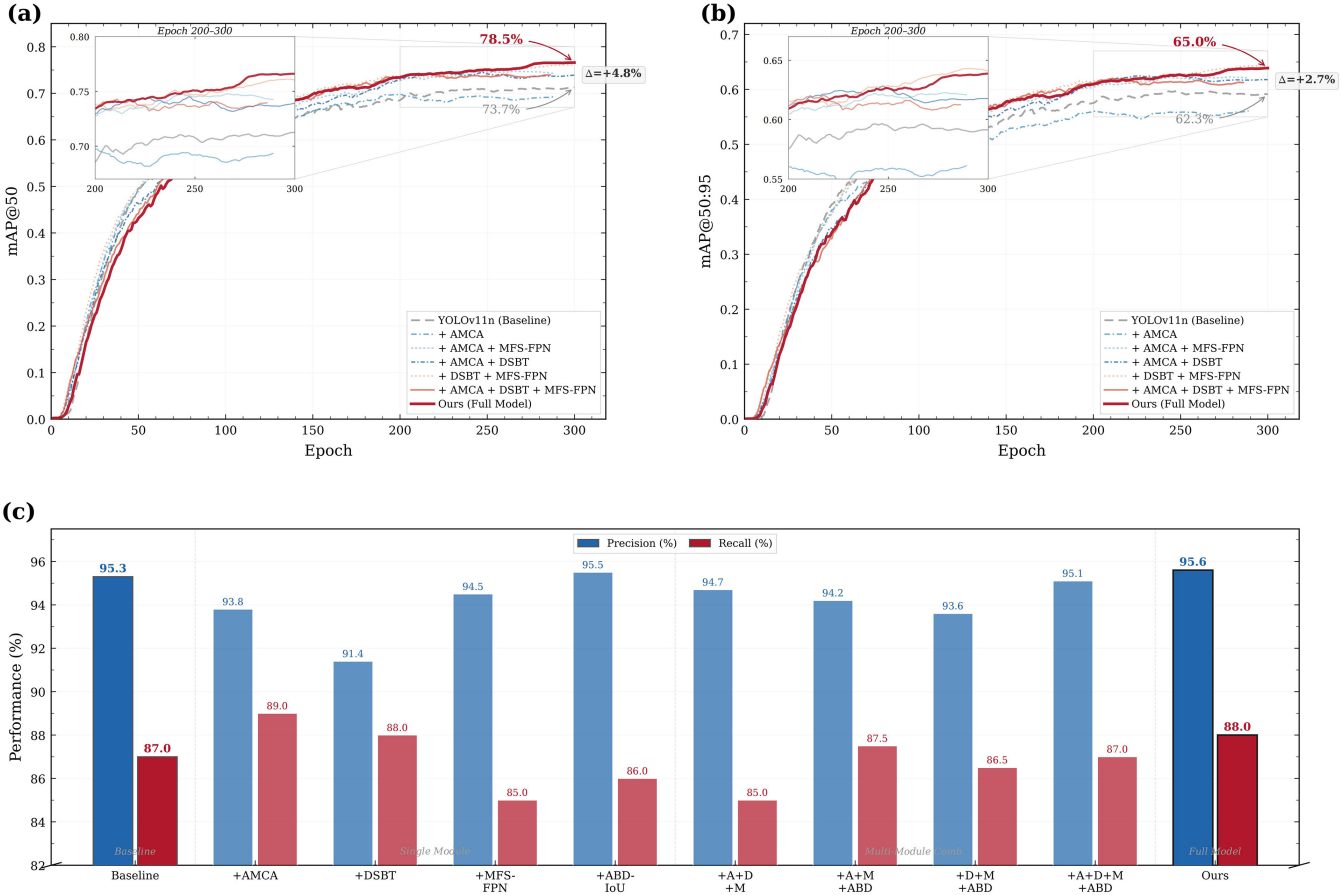
Quantitative visualization of the progressive ablation study on the CCLD dataset. **(a)** mAP@50 over 300 training epochs for the progressive ablation configurations, with the full model reaching 78.50%. **(b)** mAP@50:95 over the same epochs, with the full model reaching 65.00%. **(c)** Precision and recall for each ablation configuration, where the full model attains the highest precision (95.60%) and recall (88.00%).

A progressive ablation study on CCLD quantifies both the independent contribution of each module and cross-module interactions (**Table 5**, **Figure 6**); component-level ablations for the individual modules are provided in **Tables S1**–**S5** and **S7**. Adding any single module yields positive gains: MFS-FPN contributes the largest single-module gain in mAP@50 (+1.30%), while the ABD-IoU gain concentrates on mAP@50:95, consistent with its design goal of refining high-IoU predictions. Among two-module combinations, AMCA + DSBT achieves 76.20% mAP@50, exceeding the sum of individual contributions by 0.50%. This synergy indicates that the high-frequency response enhancement from AMCA provides higher-SNR input for boundary-prior extraction in DSBT. When all three feature modules are combined without ABD-IoU, mAP@50 reaches 77.00%, yet mAP@50:95 is 62.70%—only 0.40% above the baseline. Notably, this value is lower than the 63.80% achieved by the AMCA + DSBT pair, suggesting that adding MFS-FPN without boundary-decoupled regression shifts the optimization toward recall rather than localization precision. This pattern indicates that feature-level enhancements primarily boost recall, whereas localization accuracy under stricter IoU thresholds cannot improve proportionally without independent four-boundary decoupling in the regression stage. Adding ABD-IoU raises mAP@50:95 from 62.70% to 65.00%, confirming that boundary-decoupled regression is essential for localization accuracy; a comparison against nine mainstream IoU-family losses under the same model is provided in Table S15. Feature enhancement and regression optimization thus address complementary dimensions of performance. The full model achieves 78.50% mAP@50 (+4.80% over the baseline), 65.00% mAP@50:95, and 35.10% APS.

**Table 5:** Progressive ablation study on the CCLD dataset.

Dataset	Baseline	Components				Detection performance (%)							Δ	Speed
	YOLOv11n	AMCA	DSBT	MFS-FPN	ABD-IoU	P	R	mAP@50	mAP@50:95	APS	Param (M)	GFLOPs	mAP@50	FPS
1CCLD	✓	×	×	×	×	**95.30**	**87.00**	**73.70**	**62.30**	**28.40**	**2.60**	**6.30**	**—**	**218**
	✓	✓	×	×	×	93.80	89.00	74.80	62.80	29.10	2.35	6.20	+1.10	215
	✓	×	✓	×	×	91.40	88.00	74.60	62.60	30.50	2.70	6.31	+0.90	210
	✓	×	×	✓	×	94.50	85.00	75.00	63.50	31.20	2.61	6.33	+1.30	208
	✓	×	×	×	✓	95.50	86.00	74.20	63.10	29.80	2.60	6.30	+0.50	216
	✓	✓	✓	×	×	94.70	85.00	76.20	63.80	32.60	2.62	6.25	+2.50	211
	✓	✓	×	✓	×	94.20	87.50	76.40	63.60	32.00	2.63	6.24	+2.70	207
	✓	✓	✓	×	✓	95.00	86.50	76.60	64.60	33.20	2.62	6.25	+2.90	211
	✓		✓	✓	✓	93.60	87.50	76.80	63.70	33.00	2.72	6.34	+3.10	204
	✓	✓	✓	✓	×	95.10	87.00	77.00	62.70	34.20	2.73	6.36	+3.30	205
	✓	✓	✓	✓	✓	**95.60**	**88.00**	**78.50**	**65.00**	**35.10**	**2.73**	**6.36**	+**4.80**	**202**

Bold values indicate the best result in each column.

#### 3.2.2 Grad-CAM visualization of the overall ablation study

**Figure 7:**
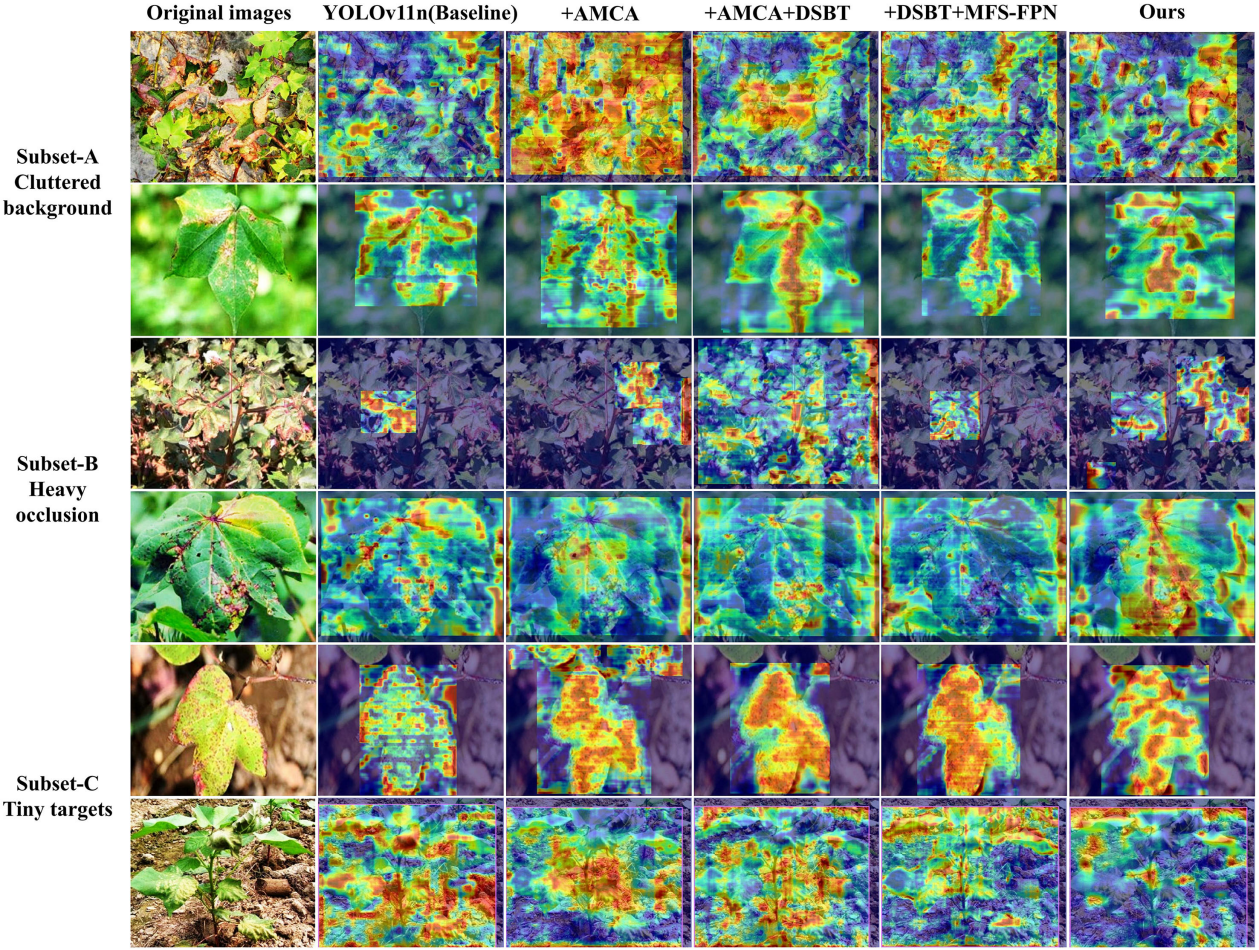
Grad-CAM visualization of the progressive ablation study under three challenge scenarios on the CCLD dataset.

Grad-CAM heatmaps visualize how each module shifts feature focus across three categories of challenging scenes (**Figure 7**). In the baseline YOLOv11, responses under cluttered backgrounds (Subset-A) are diffuse, with substantial attention scattered over irrelevant regions. Under occlusion (Subset-B) and tiny-target (Subset-C) conditions, activation over diseased areas is weak and boundary definition is poor. Adding AMCA strengthens the response over lesion regions, yet background interference remains only partially suppressed. Stacking DSBT further sharpens focus in occluded scenes, producing clearer separation between diseased and background areas. Incorporating MFS-FPN then concentrates activation over tiny targets and yields more distinct boundary contours. Across all three scene types, the full model exhibits the most concentrated heatmap distribution: high-response regions align closely with lesion locations, while background responses are visibly reduced.

#### 3.2.3 Scenario-specific performance analysis

To quantify the targeted contribution of each module to the three core challenge scenarios, the CCLD test set is partitioned by scene type (**Table 6**): Subset-A (difficult backgrounds), Subset-B (heavy occlusion), and Subset-C (tiny targets). The gain distribution across modules is broadly consistent with intended design roles. AMCA yields the largest improvement on Subset-A, where high-frequency contrast enhancement acts most directly against visually homogeneous background interference. DSBT contributes 4.40% on Subset-C and 2.90% on Subset-B, far exceeding its 1.30% gain on the background subset—evidence that cross-layer injection of shallow boundary priors is particularly critical for localizing occluded and tiny targets. Among all modules, MFS-FPN produces the largest single-module gain on Subset-C; frequency-aware channel gating mitigates the signal collapse that extremely small lesions suffer during multi-stage downsampling. The full model achieves the highest gain across all three scene types, reaching 11.80% on Subset-C, confirming that the joint benefit of all modules is most pronounced in tiny-target scenarios.

**Table 6:** Scenario-specific performance analysis on the CCLD dataset.

Model configuration	Overall mAP@50	Subset-A mAP@50	Subset-B mAP@50	Subset-C mAP@50	∆ mAP@50
**YOLOv11n Baseline**	73.70	71.20	68.50	52.30	—
+ AMCA	74.80	74.10	69.30	53.80	+1.10
+ DSBT	74.60	72.50	71.40	56.70	+0.90
+ MFS-FPN	75.00	73.80	70.90	58.40	+1.30
+ ABD-IoU	74.20	72.40	70.10	53.90	+0.50
**Full Model (Ours)**	**78.50**	**78.20**	**75.60**	**64.10**	**+4.80**

Subset-C denotes the challenge subset containing very small lesion instances, evaluated using overall mAP@50 across all size categories within this subset. This differs from AP_S_ in **Table 5**, which follows the COCO area-based definition (object area *<* 32^2^ pixels) applied to the full test set.

Bold values indicate the best result in each column.

### 3.3 Comparison with existing methods

#### 3.3.1 Comparison with existing methods on three benchmark datasets

**Figure 8:**
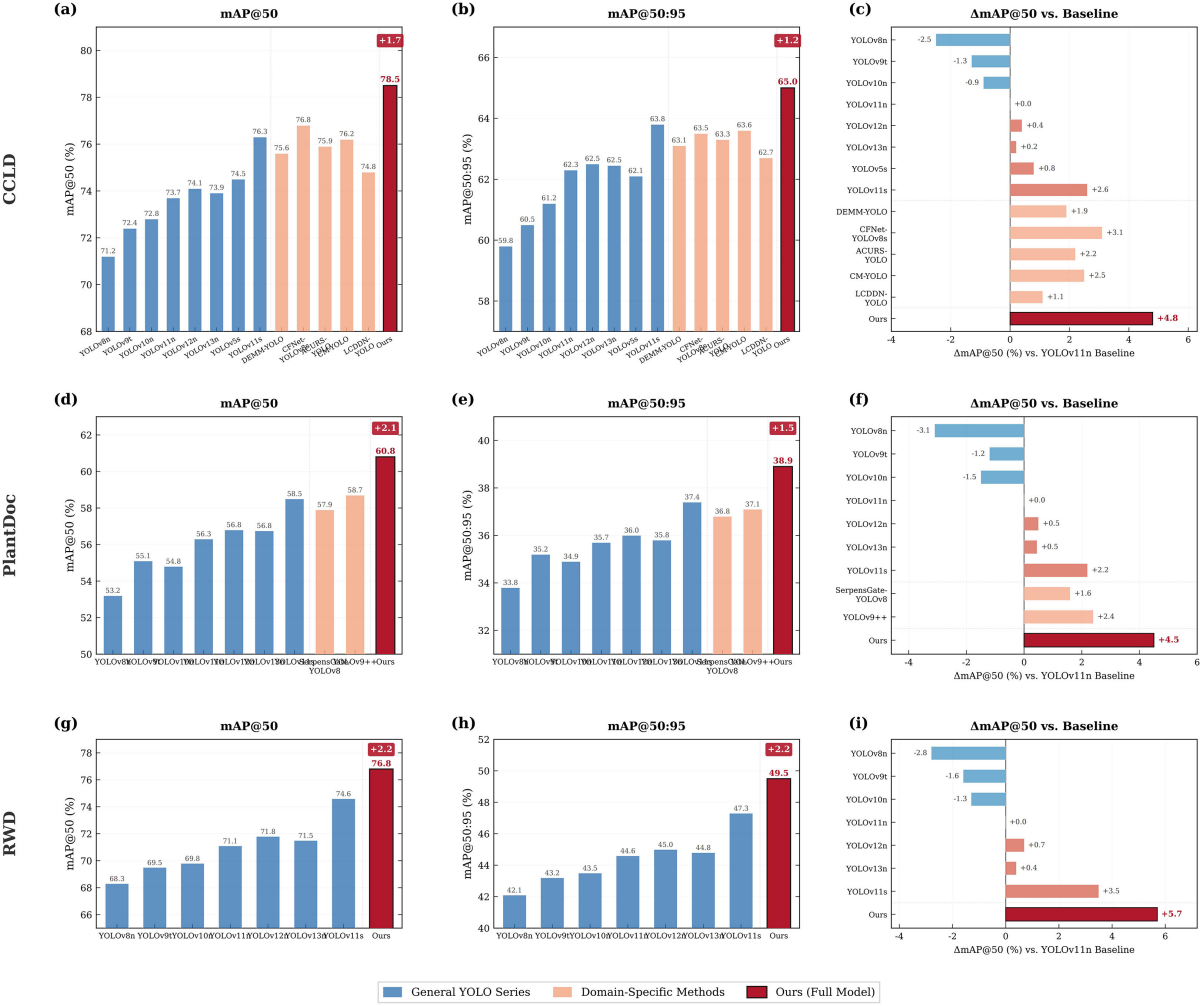
Performance comparison with existing methods on three benchmark datasets. Rows correspond to the CCLD, PlantDoc and RWD datasets. **(a, d, g)** mAP@50 and **(b, e, h)** mAP@50:95 for each method on the three datasets, with the general YOLO series in blue, domain-specific methods in orange and the proposed method in dark red. **(c, f, i)** ΔmAP@50 relative to the YOLOv11n baseline for each dataset. All values are percentages.

The proposed method is compared systematically with general-purpose YOLO baselines and domain-specific variants on CCLD, PlantDoc, and RWD to assess both detection accuracy and generalization capability. The proposed method achieves 78.50% mAP@50 on CCLD (**Table 2**; **Figure 8**) with only 2.73 M parameters, surpassing the best nano-scale model YOLOv12n by 4.40% and exceeding CFNet-YOLOv8s (11.10 M parameters) by 1.70%. For cross-domain transfer, mAP@50 reaches 60.80% on PlantDoc (+4.50%) and 76.80% on RWD (+5.70%), while inference throughput holds at 202 FPS across all three datasets. APS rises from the baseline of 28.40% to 35.10%, indicating that AMCA, DSBT, and MFS-FPN together yield complementary gains for tiny-target detection.

#### 3.3.2 Deployment feasibility analysis on edge platforms

To assess the deployability of the proposed framework on resource-constrained hardware, we benchmarked the framework on three NVIDIA Jetson platforms with TensorRT 8.6 acceleration (**Table 7**). FP32 inference matches training-time accuracy on all platforms, confirming that the AMCA re-parameterization fold — which losslessly absorbs non-standard branches into standard 3×3 kernels — causes no accuracy degradation. On Jetson Orin NX, for example, FP16 reduces latency from 12.80 ms to 7.20 ms with only a 0.20% drop in mAP@50:95, and INT8 further lowers per-inference energy consumption from the FP32 baseline of 192.0 mJ to 66.2 mJ while keeping accuracy loss within 0.90%. On the most compute-limited Orin Nano, the INT8 configuration still runs at 86 FPS with a 0.7 GB peak memory footprint and only 1.10% accuracy loss—well above the 10 FPS real-time floor for agricultural UAV operation. Differences in absolute throughput, power, and memory between the three Jetson platforms broadly track each device’s design envelope rather than properties of the model itself — Orin Nano operates within a tighter power and memory budget at the cost of throughput, while AGX Orin sits at the opposite end.

**Table 7:** Deployment performance and quantization accuracy on NVIDIA Jetson embedded platforms under TensorRT 8.6 optimization in MAX power mode.

Platform	Precision	Latency (ms)	FPS	Power (W)	Energy/Inf (mJ)	mAP @50	mAP @50:95	∆mAP @50:95	Peak memory usage
T4 (Training)	FP32	4.95	202	70.0	346.5	78.50	65.00	—	1.8 GB
Jetson Orin NX	FP32	12.80	78	15.0	192.0	78.50	65.00	0.00	1.6 GB
Jetson Orin NX	FP16	7.20	139	14.5	104.4	78.30	64.80	-0.20	1.2 GB
Jetson Orin NX	INT8	4.80	208	13.8	66.2	77.60	64.10	-0.90	0.9 GB
Jetson AGX Orin	FP32	8.50	118	30.0	255.0	78.50	65.00	0.00	1.6 GB
Jetson AGX Orin	FP16	4.90	204	28.0	137.2	78.40	64.90	-0.10	1.2 GB
Jetson AGX Orin	INT8	3.20	312	26.5	84.8	77.80	64.30	-0.70	0.9 GB
Jetson Orin Nano	FP16	18.50	54	10.0	185.0	78.20	64.70	-0.30	1.0 GB
Jetson Orin Nano	INT8	11.60	86	9.5	110.2	77.40	63.90	-1.10	0.7 GB

At inference time, AMCA folds all training-time branches into standard 3 × 3 kernels via structural re-parameterization. The resulting operator graph is identical to that of the unmodified YOLOv11n backbone. The FP32 latency reported on the T4 GPU therefore reflects the fully folded model; the small residual difference relative to the baseline arises solely from the additional operations introduced by DSBT and MFS-FPN in the neck.

These results confirm that the lightweight design is readily deployable across the tested edge platforms.

### 3.4 Cross-domain generalization

#### 3.4.1 Cross-scene transfer on PlantDoc

To assess whether the feature representations learned on CCLD generalize across scenes, this section presents two independent experiments; a cross-dataset progressive ablation replicating the CCLD protocol on PlantDoc and RWD is reported in **Table S16**. **Table 3** reports results from applying CCLD-trained models directly to three PlantDoc superclasses without fine-tuning. The proposed method achieves a mean mAP@50 of 35.80%, outperforming YOLOv11n by 4.60%. CM-YOLO, a cotton-specific detector, scores 0.73% below the general-purpose baseline on this cross-domain task, suggesting that excessive domain specialization can sacrifice generalization. **Table 8** presents the few-shot fine-tuning results. With only five annotated images per class, the proposed method reaches 42.80%, exceeding YOLOv11s given ten images. This advantage persists as the annotation budget grows to 20 images per class, where the proposed method maintains a stable lead of 6.60%.

**Table 8:** Few-shot fine-tuning on PlantDoc. K denotes the number of labeled samples per class.

Method	K=5 mAP@50	K=10 mAP@50	K=20 mAP@50	Full mAP@50
YOLOv11n	33.50	39.20	44.80	56.30
YOLOv11s	36.80	42.10	47.50	58.50
Ours	42.80	48.90	54.10	60.80

#### 3.4.2 Cross-crop transfer on RWD

To evaluate transferability on a single unseen crop, the CCLD-trained model is applied directly to the wheat disease dataset RWD without fine-tuning. The proposed method achieves mAP@50 of 38.60% (**Table 4**), surpassing the YOLOv11n baseline by 7.70% and the SPD-Conv-augmented baseline by 6.20%. These gains indicate that the high-frequency boundary preservation provided by AMCA and DSBT generalizes across leaf vascular architectures, from the broad reticulate venation of cotton to the narrow parallel venation of wheat. APS rises from 8.30% to 14.20%, an absolute gain of 5.90%, further confirming that frequency-aware channel gating transfers effectively to small-object cross-domain settings.

The 38.20% gap between zero-shot transfer accuracy and training-from-scratch accuracy reflects the inherent domain gap and dataset-scale disparity between cotton and wheat. Even under this extreme setting, however, the proposed method retains a clear advantage over competing methods, indicating that each module delivers consistent gains over the baseline across different crop morphologies.

### 3.5 Qualitative analysis

#### 3.5.1 Detection visualization under three challenge scenarios

**Figure 9:**
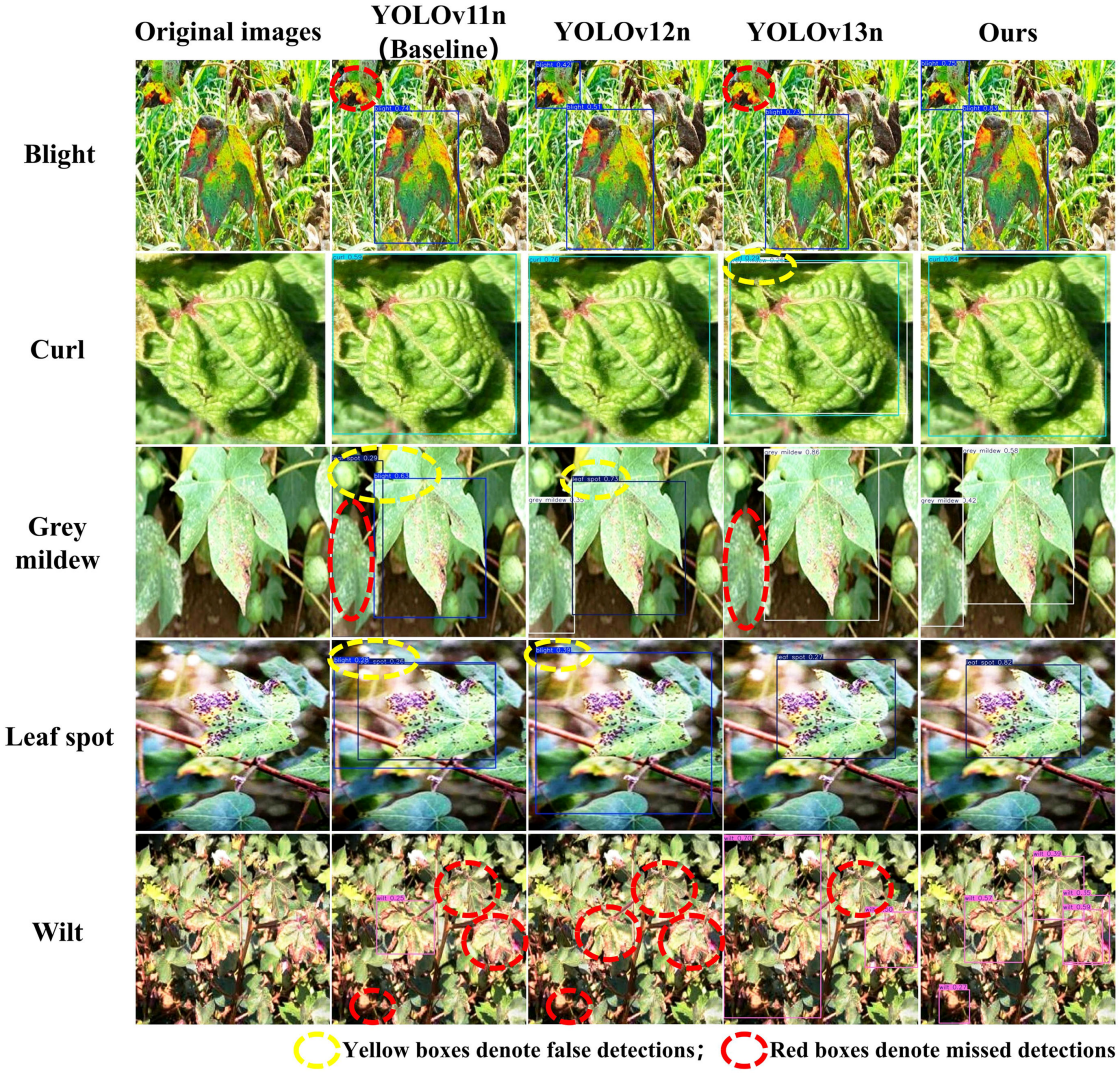
Qualitative detection comparison on five representative CCLD test images covering blight, curl, grey mildew, leaf spot, and wilt.

To qualitatively assess detection across disease categories, five representative classes—including wilt and grey mildew—are selected for visual comparison (**Figure 9**). YOLOv11n and YOLOv12n produce frequent missed detections in grey mildew scenes with complex backgrounds and in wilt scenes with densely packed small targets; they also generate false positives on leaf curl and leaf spot samples. YOLOv13n suppresses some of these false positives yet still fails to recall the missed targets in grey mildew and wilt. By contrast, the proposed method yields more accurate localization with fewer false positives and missed detections across all five classes (**Figure S6**). In the wilt scenes where small lesions are densely distributed, all three competing methods miss targets to varying degrees, whereas the proposed method detects every instance correctly. This result confirms that the proposed modules meaningfully improve both perceptual sensitivity and localization accuracy for disease-specific features.

#### 3.5.2 Cross-dataset grad-CAM comparison

**Figure 10:**
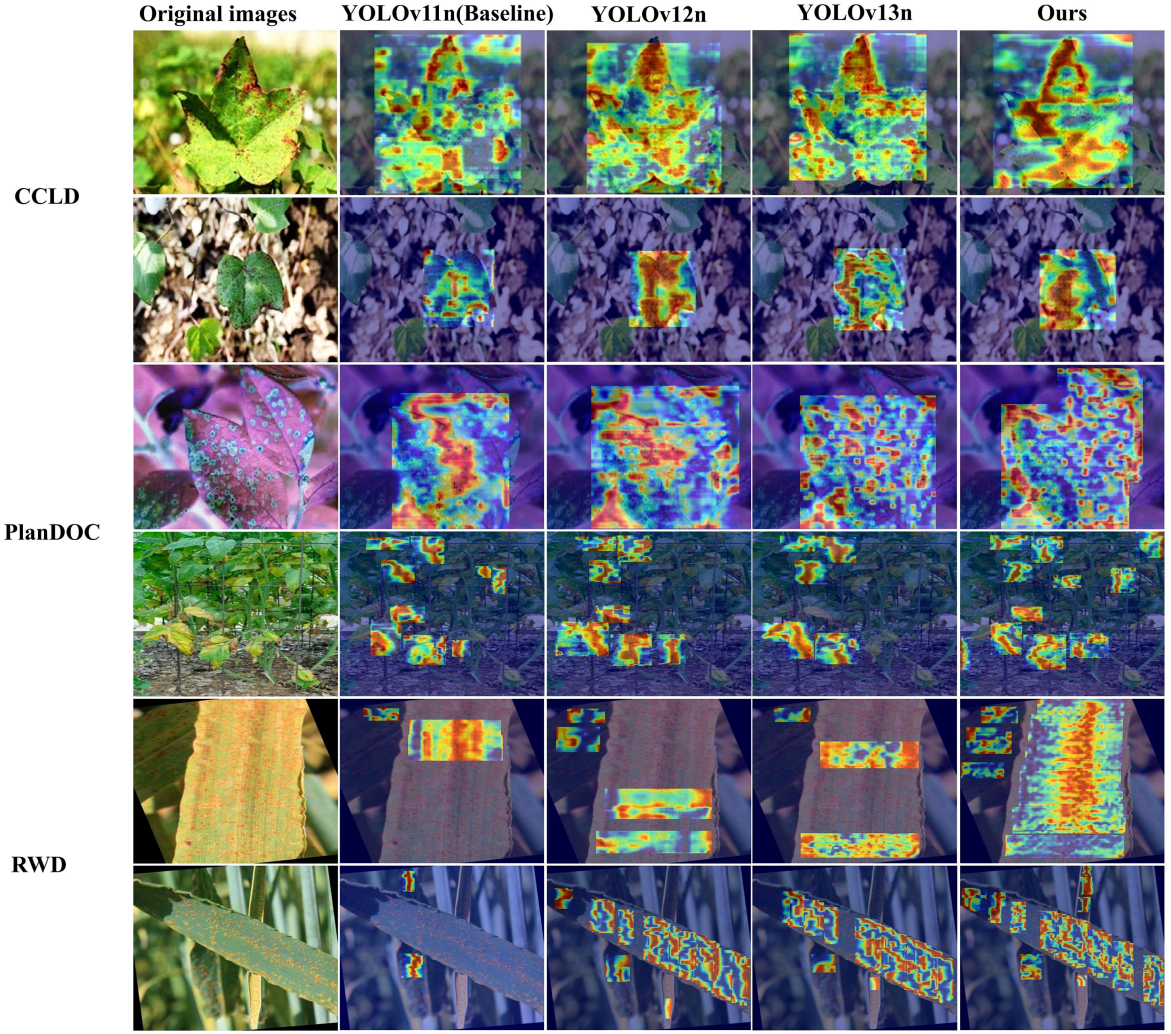
Cross-dataset Grad-CAM comparison among YOLOv11n, YOLOv12n, YOLOv13n, and the proposed method on CCLD, PlantDoc, and RWD.

To evaluate whether learned feature representations generalize across domains, this section compares Grad-CAM heatmaps on the CCLD, PlantDoc, and RWD datasets (**Figure S5**). The baseline model, YOLOv12n, and YOLOv13n all exhibit pronounced attention diffusion when confronted with unseen complex backgrounds or non-target crop structures (**Figure 10**), erroneously activating irrelevant high-frequency environmental noise. In contrast, the proposed method produces more concentrated heatmap distributions on all three datasets, with high-response regions falling closer to pathologically relevant areas. This indicates that the proposed framework captures cross-species high-frequency pathological morphology rather than merely fitting cotton-leaf-specific textures, thereby resisting attention diffusion during cross-crop transfer without finetuning.

## 4 Discussion

Combining direction-sensitive feature extraction with spatial-domain detail enhancement addresses a core limitation of general-purpose isotropic detectors: poor localization of irregular plant lesions. Experiments on CCLD show that standard square convolutional kernels and coupled bounding-box regression losses constrain localization accuracy for lesions that propagate along vascular bundles. AMCA introduces directional pathways into feature extraction through orthogonal strip convolutions, while ABD-IoU decomposes the regression penalty across four independent boundaries, allowing gradients to concentrate on whichever boundary has the largest deviation. MFS-FPN uses local mean pooling and spatial differencing to approximately separate high- and low-frequency components in the spatial domain, distinguishing high-frequency pathological signals from the background at low computational cost. The 11.80% AP gain on Subset-C (small-target scenes) aligns with this design: the central difficulty of early-stage tiny lesions is precisely that their high-frequency signals are overwhelmed by the visually homogeneous background.

On PlantDoc and RWD, zero-shot cross-domain transfer yields absolute accuracies of 35.80% and 38.60%, leaving substantial gaps to the 60.80% and 76.80% achieved by training from scratch on each dataset. These gaps reflect the inherent domain disparity between cotton and the other crops evaluated. Nevertheless, the proposed method maintains consistent relative gains over the baseline across all tested cross-domain settings: +4.60% on PlantDoc and +7.70% on RWD. These gains indicate that the direction-sensitive and boundary-aware representations reinforced by AMCA and DSBT carry transferable value under the evaluated conditions. CM-YOLO, designed specifically for cotton, falls below the general-purpose baseline in cross-domain transfer, suggesting that excessive domain specialization may sacrifice generalization. That said, the absolute accuracy of zero-shot transfer remains insufficient for practical cross-crop deployment; fine-tuning with a small number of target-domain annotations is still required. The cross-domain comparisons on PlantDoc and RWD are conducted primarily against general-purpose baselines, because most existing domain-specific methods have not released code or adopt different experimental protocols that preclude direct inclusion.

The 4.80% mAP@50 improvement on CCLD should be interpreted with care in the context of real agricultural scenarios. For UAV-based scouting systems, the gains matter in three respects. First, improved recall of early-stage tiny lesions (11.80% gain on Subset-C) can advance the disease detection window. Second, the improvement under heavy occlusion (7.10% gain on Subset-B) reduces miss rates in densely planted areas. Third, the inference speed of 202 FPS on edge platforms meets real-time processing requirements. However, the scale of CCLD (6,856 images) and its geographic coverage (restricted to Xinjiang cotton fields) impose objective limits on the generalizability of these conclusions. Although the annotation consistency metrics — mean pairwise IoU of 0.82 ± 0.07 and Fleiss’ *κ* of 0.78— indicate substantial agreement, they fall slightly below the 0.85+ threshold common in rigid-object datasets. This implies an upper bound of roughly 1–2% annotation noise in the evaluation metrics, a factor that should be considered when interpreting small margins in the ablation study.

Three specific limitations apply to the current results. First, the approximation of high-frequency energy by spatial variance in MFS-FPN relies on adequate suppression of low-frequency background by AMCA; removing AMCA causes the Pearson r to drop from 0.83 to 0.52. When the background itself exhibits high spatial variance—e.g., dappled light and shadow—this approximation may become unreliable. Second, ABD-IoU removes the aspect-ratio penalty term present in CIoU. Although this removal resolves the conflict with four-boundary independent decoupling, the absence of a global shape constraint may cause predicted boxes to take unreasonable shapes under severe occlusion, where visible boundary information is extremely limited. This concern is not directly tested in the current work and constitutes an open question for future investigation. Third, the entire framework operates on visible-light RGB imagery. For infections still in the latent period that have not yet produced macroscopically visible symptoms, a purely morphological and spatial-domain high-frequency feature extraction approach faces a fundamental perceptual bottleneck (**Figure S7**).

Two engineering trade-offs also merit discussion. DCNv2 achieves a slightly higher AP in the diagonal direction (+3.30%) but reduces inference throughput to 148 FPS, violating the edge deployment constraint (**Table S8**). Similarly, the spatial-domain statistical approximation in MFS-FPN is less accurate than FFT but remains compatible with embedded platforms that lack native FFT hardware support—a trade-off driven directly by deployment requirements.

Overall, the 4.80% mAP@50 improvement on CCLD, the 11.80% gain on tiny-lesion scenes, and the consistent +4.50%–5.70% relative improvements across two cross-domain datasets jointly exceed the per-paper gain typically reported by recent cotton/plant disease detectors (e.g., +1.10%–3.10% in LCDDN-YOLO, DEMM-YOLO, ACURS-YOLO, CM-YOLO and CFNet-YOLOv8s under comparable baselines), while using only 2.73 M parameters and maintaining 202 FPS. These improvements span coarse and strict IoU thresholds, object scales, deployment precisions, and cross-domain conditions.

## 5 Conclusions

This work shows that the anisotropic propagation of cotton lesions, shaped by vascular constraints, can serve as a prior for feature adaptation and boundary optimization in detection models. Organized around the principle of preserving high-frequency boundary information across the pipeline, AMCA establishes directional coverage and extracts high-frequency residuals at the feature front end; DSBT transfers boundary priors before downsampling; and MFS-FPN retains detail channels in the neck through variance-aware gating. At the regression stage, ABD-IoU applies independent penalties to four physical boundaries. Together, the four modules span the complete pipeline from feature extraction to coordinate regression. In the ablation study, the joint AMCA–DSBT configuration yields a gain 0.50% above the sum of their individual contributions, confirming inter-module synergy rather than simple additive stacking.

On CCLD, the proposed method achieves 78.50% mAP@50 (+4.80%) and 65.00% mAP@50:95 (+2.70%) with 2.73 M parameters at 202 FPS. The model runs in real time on a Jetson Orin NX under INT8 quantization. In cross-domain evaluation, relative gains reach +4.50% on PlantDoc and +5.70% on RWD. A clear gap nevertheless remains between zero-shot transfer accuracy and training from scratch, and cross-crop deployment still requires fine-tuning with a small number of target-domain annotations.

At the engineering level, structural re-parameterization folds all training-time multi-branch topologies into standard 3 × 3 kernels at inference time, and spatial-domain variance statistics replace dependence on FFT hardware. Together, these two designs ensure that direction-sensitive and frequency-aware capabilities incur no additional inference overhead. ABD-IoU refines gradient attribution of the regression loss from a two-dimensional width–height formulation to one-dimensional granularity over four independent boundaries—left, right, top, and bottom. This decoupling strategy may also apply to other agricultural targets that exhibit single-side offset, but its effectiveness beyond cotton remains to be validated experimentally.

The current framework processes only visible-light RGB imagery and therefore cannot detect latent-period infections. The geographic coverage of CCLD is restricted to Xinjiang cotton fields, and whether these conclusions generalize to other production regions requires further investigation. Two directions are planned. The first is to incorporate near-infrared or multispectral bands, exploiting spectral response differences between biotic stress and physical damage in non-visible wavelengths to resolve the visual ambiguity between the two. The second is to augment ABD-IoU with a lightweight adaptive shape regularization term that provides global geometric constraints on predicted boxes under extreme occlusion.

## Data Availability

The datasets presented in this study can be found in online repositories. The names of the repository/repositories and accession number(s) can be found below: https://github.com/DynaVLA/ABAD-CLD.
