# Supplementary material for: Anisotropic boundary-aware detection for cotton leaf diseases with boundary-decoupled regression and lightweight feature adaptation
Source: Front Plant Sci. 2026 Jul 15;17:1848014. doi: 10.3389/fpls.2026.1848014 (PMC13415594; doi:10.3389/fpls.2026.1848014)
Supplement: Supplementary file 1 [file DataSheet1.pdf]

Appendix

S1 Dataset Visualization and Geometric Analysis

Figure S1 illustrates the dual challenges the CCLD dataset presents: visual appearance and geometric morphology. Figure S1a highlights three difficult scenarios—visually homogeneous background interference (Subset-A), heavy occlusion (Subset-B), and tiny early-stage lesions (Subset-C)—all of which demand strong high-frequency feature extraction. Figure S1b shows that bounding-box aspect ratios exhibit a wide, markedly asymmetric distribution, reflecting anisotropic lesion propagation driven by vascular constraint. This high-variance geometric profile exposes the limitation of conventional isotropic regression losses under extreme boundary offsets and directly motivates the anisotropic boundary-decoupled penalty (ABD-IoU) introduced in this work.

S2 Ablation Study

S2.1 Ablation Study on the AMCA Module

Progressive ablation of the three core AMCA components (Table S1) confirms that each contributes measurably and that the two main branches interact synergistically. The standard 3×3 RepConv alone improves mAP@50 by 0.20%. Anisotropic Re-parameterizable Convolution (AnisoRepConv), which introduces 1×3 and 3×1 strip branches, raises the gain to 0.60%, indicating that directional receptive-field coverage, rather than multi-branch BN regularization, is the primary driver. HFC-Ghost alone improves mAP@50 by 0.40%, with APS reaching 28.80%—slightly above the 28.70% from AnisoRepConv—suggesting that high-frequency residual extraction contributes more directly to small-object edge detection. Combining the two yields 74.60% mAP@50 and 63.00% mAP@50:95; the latter exceeds the sum of individual contributions by 0.10%, indicating synergy between morphological adaptation and frequency-domain enhancement under strict IoU thresholds. In drop-in replacement comparisons, AnisoRepConv outperforms ACNet under all paired conditions and HFC-Ghost outperforms Standard Ghost, confirming that each design improvement contributes independently. Adding Split-Concat multi-scale aggregation, the full AMCA configuration reaches 74.80% mAP@50 and 29.10% APS with only 2.35 M parameters.

S2.2 Ablation Study on the DSBT Module

Progressive ablation of the three DSBT sub-modules (Table S2) reveals that each stage addresses a distinct aspect of boundary-aware detection. The semantic gradient operator with multiple directions alone improves mAP@50 by 0.20%, showing that the learnable gradient operator can extract meaningful boundary priors from shallow semantic features. Adding the boundary pyramid generator raises mAP@50 further to 74.20%, while mAP@50:95 remains at 62.40%. This divergence indicates that multi-scale boundary priors help the model detect more objects under a loose IoU threshold, yet the localization of these additional detections is not precise enough to benefit under strict thresholds—underscoring the need for spatial modulation in BGSM to improve localization accuracy. Replacing the learnable depthwise separable convolution downsampling in the pyramid generator with MaxPool reduces mAP@50 to 74.00% and mAP@50:95 to 62.35%, confirming that strided convolution preserves sub-pixel boundary displacement information better than fixed pooling. Incorporating spatial modulation guided by boundary cues then raises mAP@50 to 74.60% and mAP@50:95 to 62.60%, converting boundary priors into localization gains through spatial multiplicative modulation. The entire DSBT mechanism adds only 0.10 M parameters and 0.01 GFLOPs.

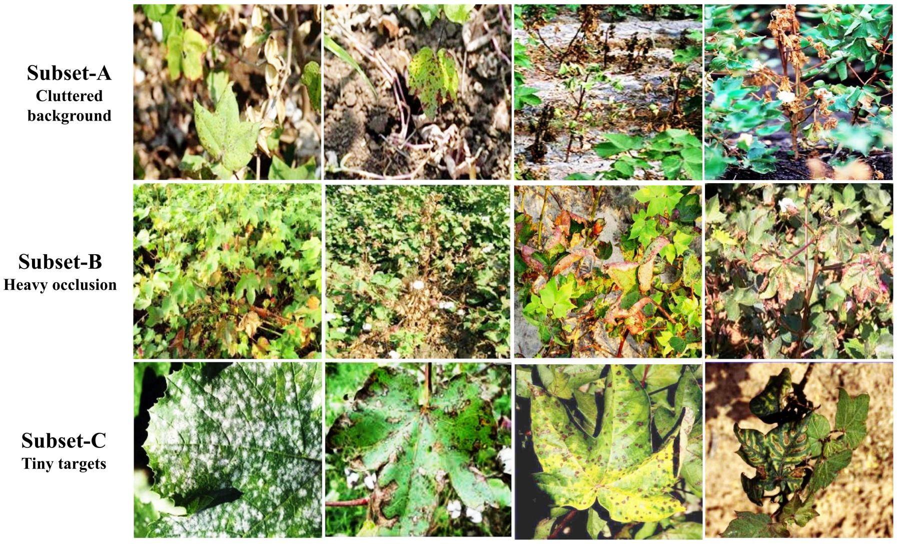

(a) Representative samples from the three challenge subsets of the CCLD dataset.

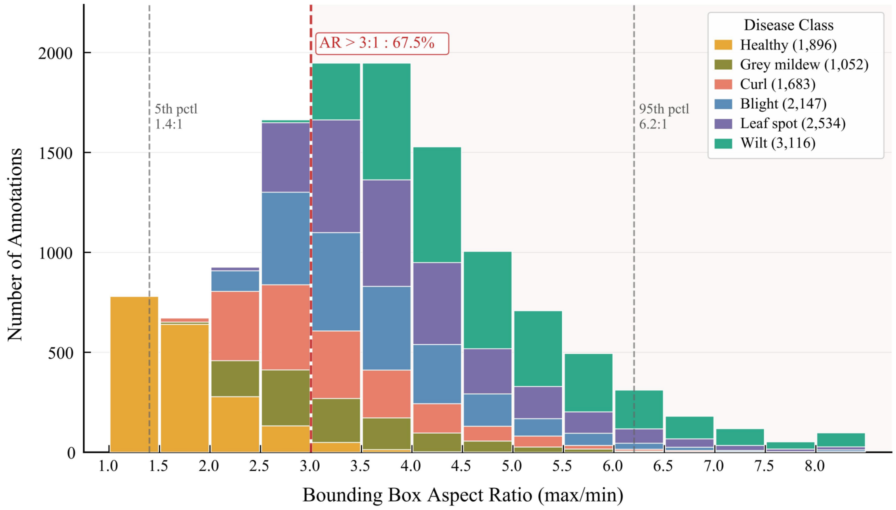

(b) Distribution of bounding-box aspect ratios across disease classes in the CCLD dataset.

Figure S1: Dataset visualization and geometric analysis of the CCLD dataset.

Table S1: Component-level ablation of the AMCA module on the CCLD dataset

Model Configuration	Aniso-RepConv	HFC-Ghost	Split-Concat	mAP @50	mAP @50:95	Param(M)	GFLOPs	AP_S
**YOLOv11n Baseline**	×	×	×	**73.70**	**62.30**	**2.60**	**6.30**	**28.40**
+ Standard RepConv (3 × 3 only)	✓(3 × 3)	×	×	73.90	62.40	2.25	6.22	28.50
+ AnisoRepConv (1 × 3 + 3 × 1 + 3 × 3)	✓	×	×	74.30	62.70	2.22	6.21	28.70
+ HFC-Ghost only	×	✓	×	74.10	62.50	2.18	6.18	28.80
+ AnisoRepConv + HFC-Ghost	✓	✓	×	74.60	63.00	2.19	6.19	29.00
ACNet (1 × 3 + 3 × 1 + 3 × 3) + Standard Ghost	✓ (ACNet)	× (Std)	×	74.20	62.70	2.20	6.19	28.80
ACNet (1 × 3 + 3 × 1 + 3 × 3) + HFC-Ghost (Ours)	✓ (ACNet)	✓	×	74.40	62.85	2.18	6.18	29.00
AnisoRepConv (Ours) + Standard Ghost	✓	× (Std)	×	74.35	62.75	2.22	6.21	28.90
**AMCA (Full)**	✓	✓	✓	**74.80**	**63.10**	**2.35**	**6.20**	**29.10**

Table S2: Sub-module ablation of the DSBT mechanism on the CCLD dataset

Configuration	DSGO	SBPG	BGSM	mAP@50	mAP@50:95	Param(M)	GFLOPs
**YOLOv11n Baseline**	×	×	×	**73.70**	**62.30**	**2.60**	**6.30**
+ DSGO only	✓	×	×	73.90	62.40	2.63	6.31
+ DSGO + SBPG	✓	✓	×	74.20	62.40	2.67	6.31
+ DSGO + SBPG	✓	MaxPool	×	74.00	62.35	2.65	6.30
**DSBT (Full)**	✓	✓	✓	**74.60**	**62.60**	**2.70**	**6.31**

S2.3 Ablation Study on the MFS-FPN Module

Progressive ablation and permutation experiments on the three MFS-FPN sub-modules show that each contributes distinctly and that their ordering materially affects performance. In the single-component comparison (Table S3), HVP-Down contributes the most, reaching 74.40% mAP@50 and 63.00% mAP@50:95; variance-aware gating preserves informative high-frequency channels more directly during dimensionality reduction. AMI-Hub captures directional morphological interactions through strip convolutions, raising mAP@50 to 74.20%. In the window-size ablation for SEFD, k = 5 and k = 7 achieve comparable accuracy while the former incurs lower computation; k = 3 degrades noticeably due to an insufficient receptive field, so k = 5 is adopted. Combining all three yields 75.00% mAP@50 and 63.50% mAP@50:95. In the full MFS-FPN configuration, replacing HVP-Down with SPD-Conv combined with a 1×1 convolution lowers accuracy while increasing both parameters and computation, confirming that selectively preserving high-frequency channels through variance-aware gating is especially critical for small-object detection, where such signals are

sparse. The permutation ablation (Table S4) shows that placing dimensionality reduction first constrains the receptive field of the strip convolutions in a compressed channel space, reducing mAP@50 by 0.50%. Placing high-/low-frequency separation first introduces redundant mean-pooling computation over the full channel space, increasing GFLOPs by 0.15 G while reducing accuracy by 0.70%. These results confirm that morphological interaction is most effective when performed in the full channel space, before channel compression and frequency separation.

Table S3: Sub-module ablation of MFS-FPN on the CCLD dataset

**Model Configuration**	**AMI-Hub**	**HVP-Down**	**SEFD**	**mAP @50**	**mAP @50:95**	**Param(M)**	**GFLOPs**
YOLOv11n Baseline (Standard Neck)	×	×	×	73.70	62.30	2.60	6.30
+ AMI-Hub only	✓	×	×	74.20	62.80	2.61	6.32
+ HVP-Down only (without AMCA)	×	✓	×	74.40	63.00	2.60	6.31
+ SEFD (k=3)	×	×	✓	73.80	62.40	2.61	6.31
+ SEFD (k=5)	×	×	✓	74.10	62.70	2.61	6.32
+ SEFD (k=7)	×	×	✓	74.10	62.70	2.61	6.33
+ AMI-Hub + HVP-Down	✓	✓	×	74.70	63.20	2.61	6.33
AMI-Hub + SPD-Conv (replacing HVP-Down) + SEFD	✓	×	✓	74.50	63.00	2.68	6.45
MFS-FPN (Full)	✓	✓	✓	75.00	63.50	2.61	6.33

Table S4: Processing order ablation for the three MFS-FPN sub-modules

Order	mAP@50	mAP@50:95	GFLOPs
AMI-Hub → HVP-Down → SEFD (Default)	75.00	63.50	6.33
HVP-Down → AMI-Hub → SEFD	74.50	63.00	6.33
SEFD → AMI-Hub → HVP-Down	74.30	62.80	6.48

S2.4 ABD-IoU Component Ablation and Temperature Sensitivity Analysis

To isolate the independent contribution of each ABD-IoU component, Table S5 holds the full feature-module configuration (AMCA + DSBT + MFS-FPN) fixed and varies only the loss function. Because DIoU is mathematically equivalent to CIoU with αv removed, and EIoU replaces αv with independent width–height penalties, the DIoU and EIoU entries from the loss-function study (Table S5) serve as direct controls. Reverting CIoU to DIoU, C1 → C2, lowers mAP@50:95 by 0.20%, indicating that αv removal alone is mildly harmful and that ABD-IoU's gains cannot be attributed to discarding the aspect-ratio constraint. Progressing from DIoU through EIoU to four-boundary

decoupling, C2 → C3 → C4, yields monotonically increasing mAP@50:95 of 62.50%, 62.90%, and 64.20%. The 1.30-point gap between 2D and 4D decoupling is the largest single increment and accounts for 56.5% of the total CIoU-to-ABD-IoU improvement, confirming that per-boundary gradient attribution—rather than αv removal or width–height separation—is the dominant mechanism.

Table S5: Component ablation of ABD-IoU loss on the CCLD dataset

#	Configuration	αv	Decoupling	LSM	mAP@50	mAP@50:95	mAP@75	AP_S	AP_M	AP_L
C1	CIoU (Baseline)	√	Coupled	×	77.00	62.70	67.40	33.20	58.40	72.10
C2	DIoU (αv removed)	×	None	×	76.80	62.50	67.10	32.80	58.10	71.80
C3	EIoU (2D W/H decoupling)	×	2D (W, H)	×	77.20	62.90	67.60	33.00	58.60	72.30
C4	4-Boundary Decoupling (no LSM)	×	4D (L,R,T,B)	×	77.80	64.20	68.50	34.50	59.60	73.20
C5	CIoU + LSM (no Decoupling)	√	Coupled	√	77.40	63.10	67.80	33.45	58.85	72.35
C6	ABD-IoU (Full)	×	4D (L,R,T,B)	√	78.50	65.00	69.20	35.10	60.20	73.80

Four-boundary decoupling and LSM exhibit super-additive interaction. Their independent gains sum to 1.90%, yet the combined configuration C6 achieves 2.30% over the CIoU baseline. The source of this surplus can be traced to the asymmetric LSM response: adding LSM to four-boundary decoupling (C4 → C6) yields 0.80%, double the 0.40% obtained on coupled CIoU (C1 → C5), indicating that LSM's gradient-sustaining effect is amplified when the underlying penalty already provides directionally precise signals. The temperature sensitivity analysis (Table S6) corroborates this high-IoU focus: mAP@90 fluctuates by up to 1.90% across τ values compared with only 0.70% for mAP@50, and $\tau = 10$ yields the best trade-off across all metrics, which is adopted as the default setting.

Table S6: Sensitivity analysis of the LSM temperature parameter τ on the CCLD dataset

τ	mAP@50	mAP@50:95	mAP@75	mAP@90
1	77.80	64.20	68.50	47.30
5	78.10	64.60	68.90	48.10
10	78.50	65.00	69.20	49.20
20	78.30	64.80	69.00	48.80
50	77.90	64.30	68.40	47.60

S2.5 Cross-Stage Complementarity between HFC-Ghost and SEFD

Four cross-experiments verify the complementarity of HFC-Ghost and SEFD in suppressing low-frequency interference at different network stages (Table S7). Each module alone improves mAP@50 by 0.40%, yet SEFD contributes 0.40% to mAP@50:95 compared with 0.20% from HFC-Ghost, indicating that high-/low-frequency separation at the neck—closer to the detection head—is more effective at improving boundary accuracy under strict IoU thresholds. Combining the two produces gains that exceed the sum of individual contributions, confirming that high-frequency residual extraction in the backbone and low-frequency suppression in the neck play complementary roles.

S2.6 AnisoRepConv Directional Coverage Analysis

A direction-grouped AP comparison (Table S8) disentangles the respective contributions of directional receptive-field expansion and multi-branch regularization. Multi-3×3 RepConv, which

Table S7: Cross-stage complementarity between HFC-Ghost (backbone) and SEFD (neck) for high-frequency signal preservation on the CCLD dataset.

#	HFC-Ghost (Backbone)	SEFD (Neck)	mAP@50	mAP@50:95
1	×	×	73.70	62.30
2	√	×	74.10	62.50
3	×	√	74.10	62.70
4	√	√	74.60	63.10

matches AnisoRepConv in both branch count and BN count, improves overall mAP@50 by only 0.20%, whereas AnisoRepConv improves it by 1.10%, with horizontal and vertical directions gaining 3.70% and 4.30%, respectively. This gap confirms that the gain stems primarily from orthogonal strip branches capturing the directional features of lesions propagating along veins, rather than from multi-branch training regularization. Compared with DCNv2, AnisoRepConv is 0.30% and 0.40% higher in the horizontal and vertical directions but 3.30% lower in the diagonal direction, reflecting the inherent limitation of orthogonal strip kernels in covering non-axial targets. The overall mAP@50 of DCNv2 is only 0.50% higher, however, at the cost of reducing inference throughput from 215 FPS to 148 FPS; its irregular memory-access patterns degrade TensorRT efficiency, violating edge-deployment constraints. AnisoRepConv, by contrast, folds entirely into a standard 3×3 kernel at inference time, introducing no additional overhead.

Table S8: Orientation-grouped AP@50 (%) analysis on the CCLD dataset

Method	Horizontal AP	Vertical AP	Diagonal AP	Overall mAP	FPS
YOLOv11n (3×3)	72.80	71.50	68.30	73.70	218
+ Multi-3×3 RepConv (same BN count)	73.60	72.30	68.60	73.90	216
+ AnisoRepConv (1×3+3×1)	76.50	75.80	69.80	74.80	215
+ DCNv2	76.20	75.40	73.10	75.30	148

S2.7 DSGO Direction Count Ablation

An ablation from two to eight directions determines the optimal configuration for the semantic gradient operator with multiple directions (Table S9). Increasing from two to four directions improves mAP@50 by 0.40%, whereas a further increase to six directions yields only an additional 0.10% at 50% more parameters. Eight directions match six in mAP@50 exactly, while mAP@50:95 drops slightly, indicating that the marginal return of additional directional coverage saturates beyond four directions. Because parameter count scales linearly—each direction adds 1.6 K parameters—four directions represents the optimal trade-off between accuracy and efficiency.

S2.8 Frequency Response Verification of the HFC-Ghost $\text{DWConv}_{5\times5}$ Kernel

A comparison against a fixed Gaussian kernel and a no-HFC-Ghost baseline verifies whether the learnable depthwise convolution kernel in HFC-Ghost retains approximate low-pass filtering characteristics after training convergence (Table S10). The fixed low-pass kernel alone yields a 0.30% mAP@50 gain over the baseline, indicating that high-frequency residual extraction is itself an effective mechanism. The learnable kernel, at identical parameter count and computation, further raises

Table S9: Direction count ablation for the DSGO in the DSBT module

Directions	Configuration	mAP@50	mAP@50:95	Extra Params(K)
2	$\{0°, 90°\}$	74.20	62.30	3.2
4	$\{0°, 90°, 45°, 135°\}$ (Default)	74.60	62.60	6.4
6	$\{0°, 30°, 60°, 90°, 120°, 150°\}$	74.70	62.70	9.6
8	8-direction uniform	74.70	62.60	12.8

mAP@50 to 74.80% and small-object accuracy to 29.10%, showing that end-to-end optimization endows the filter with stronger frequency adaptability than a fixed Gaussian kernel.

Table S10: HFC-Ghost $\text{DWConv}_{5\times5}$ filter behavior verification on the CCLD dataset.

Configuration	Filter Type	mAP@50	mAP@50:95	AP_S	Param(M)	GFLOPs
Baseline (no HFC-Ghost)	—	73.70	62.30	28.40	2.60	6.30
HFC-Ghost w/ Fixed Gaussian (σ=1.0)	Fixed low-pass	74.00	62.40	28.90	2.35	6.20
HFC-Ghost w/ Learnable DWConv (Default)	Learned	74.80	62.80	29.10	2.35	6.20

Figure S2a presents a 2-D Fourier transform of the trained kernel weights, with the magnitude spectrum remaining heavily concentrated in the low-frequency central region. Phase-spectrum analysis reveals that the phase standard deviation among low-frequency components is 33°, well below the 104° theoretical standard deviation of a uniform random distribution, indicating phase coherence that satisfies the spatial homogeneity condition of a low-pass filter. Together, the magnitude and phase spectra provide joint frequency-domain evidence supporting the approximate low-pass hypothesis.

Figure S2b shows layer-wise feature visualizations for three representative disease morphologies. The depthwise convolution output preserves smooth low-frequency structure, while the spatial-difference residual consistently highlights high-frequency responses at lesion edges and necrotic textures—consistent with the theoretical expectation derived from the formulation.

S2.9 Variance–High-Frequency Energy Correlation Verification for HVP-Down

To verify the core assumption in HVP-Down—that channel-wise spatial variance approximates high-frequency energy—we extract feature maps at the HVP-Down input layer from the CCLD test set using forward hooks. Spatial variance and 2-D FFT-based high-frequency energy ratio are computed per channel, with the cutoff frequency set to 50% of the Nyquist frequency. Pearson r values across P3–P5 are 0.83, 0.81, and 0.78, and Spearman ρ values are 0.79, 0.77, and 0.74 (Table S11), all indicating strong positive correlation with p-values far below $1e^{-10}$.

The slight decline in correlation from shallow to deep layers (Figure S3) is expected: deeper features encode higher-level semantics, partially diluting the direct correspondence between variance and high-frequency energy, yet the correlation remains within the strong range. Removing AMCA causes r to drop sharply to 0.52, indicating that front-end suppression of low-frequency background components by AMCA markedly strengthens the reliability of variance as a high-frequency energy proxy. This finding provides an empirical foundation for the variance-aware gating design of HVP-Down.

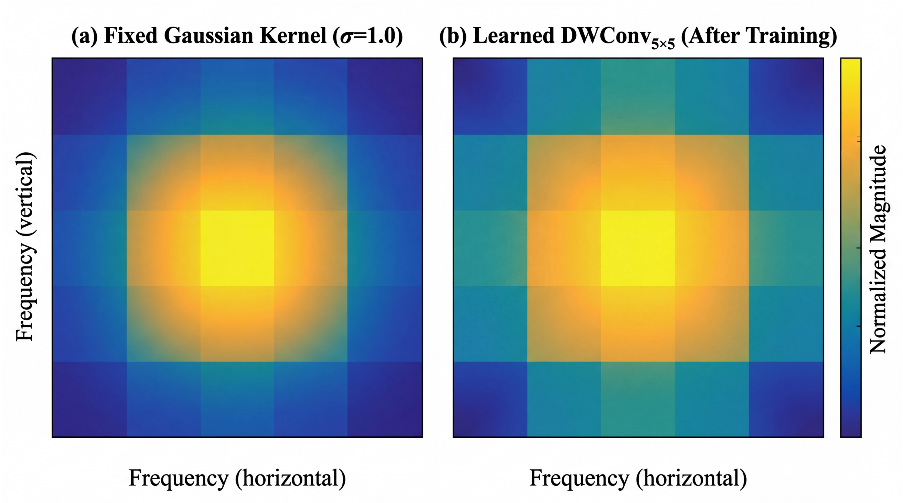

(a) Frequency-domain verification of the learned HFC-Ghost DWConv$_{5\times5}$ kernel.

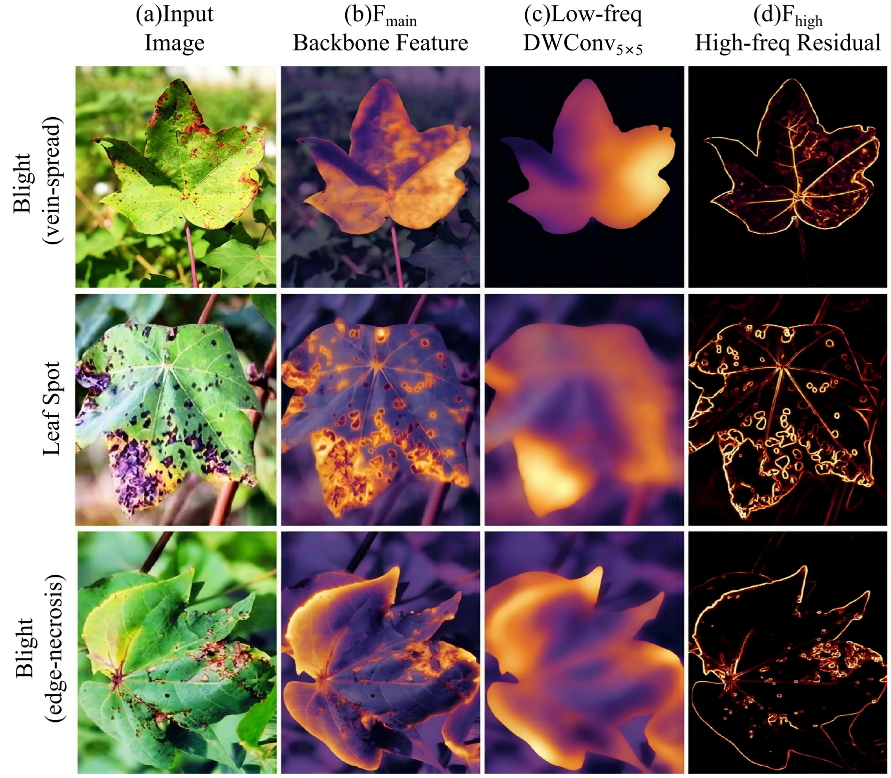

(b) Layer-wise feature visualization of HFC-Ghost for three representative disease morphologies (blight, wilt, leaf spot).

Figure S2: Frequency-domain and feature-level verification of the HFC-Ghost design.

Table S11: Correlation between channel spatial variance and high-frequency energy ratio at HVP-Down input on the CCLD test set

Measurement Point	# Channels	# Samples	Pearson r	p-value	Spearman ρ
HVP-Down input (P3 scale)	256	175,616	0.83	<1e-10	0.79
HVP-Down input (P4 scale)	512	175,104	0.81	<1e-10	0.77
HVP-Down input (P5 scale)	1024	174,080	0.78	<1e-10	0.74
Without AMCA (P3 scale, control)	256	175,616	0.52	<1e-6	0.48

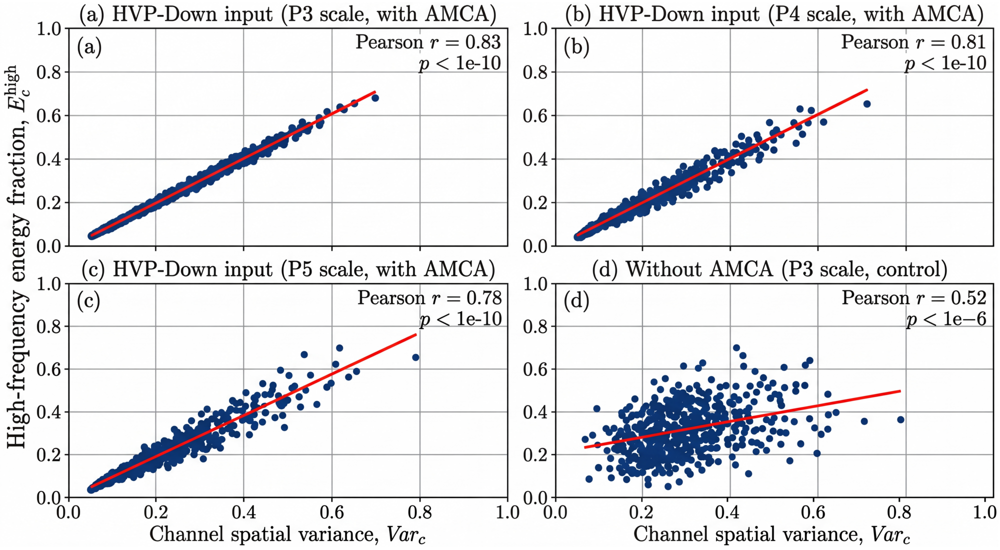

Figure S3: Scatter plots of channel-wise spatial variance vs. high-frequency energy ratio at P3, P4, and P5 scales of HVP-Down input on the CCLD test set

S3 Supplementary Results

S3.1 Statistical Significance Verification

To rule out the influence of randomness on experimental conclusions, we perform statistical significance testing over 10 independent runs with different random seeds for the proposed method and five main comparison models (Table S12). Across all five paired comparisons the Wilcoxon signed-rank test yields p = 0.002—the smallest two-sided p-value attainable for n = 10 when all 10 paired differences share the same sign—indicating that the proposed method outperforms each baseline in every run. The paired t-test returns p < 0.001 in all cases, satisfying the $\alpha = 0.01$ significance level and excluding the possibility that the observed performance gaps arise from random fluctuation. The proposed method also exhibits the lowest standard deviation among all models across the 10 runs, at ±0.35 for mAP@50 and ±0.27 for mAP@50:95, indicating that the synergistic design of the four modules does not introduce additional optimization uncertainty.

Table S12: Statistical significance verification over 10 independent runs with different random seeds on the CCLD dataset

Model	mAP@50 (Mean±Std)	mAP@50:95 (Mean±Std)	Wilcoxon p	Paired t p	Sig.
YOLOv11n	73.52±0.38	62.18±0.29	0.002	<0.001	**
YOLOv12n	73.89±0.41	62.36±0.31	0.002	<0.001	**
DEMM-YOLO	75.42±0.45	62.95±0.33	0.002	<0.001	**
ACURS-YOLO	75.71±0.43	63.14±0.35	0.002	<0.001	**
CM-YOLO	76.03±0.39	63.42±0.30	0.002	<0.001	**
**Ours**	**78.33±0.35**	**64.87±0.27**	—	—	—

S3.2 LSM Convergence and Learning Rate Schedule Dependency

Four cross-experiments on the CCLD dataset—crossing two loss functions with two learning-rate strategies—verify the dependence of the non-zero limiting gradient of LSM on the learning-rate schedule (Table S13). ABD-IoU under cosine annealing improves mAP@50 by 1.30% over a fixed learning rate, substantially greater than the 0.60% gain CIoU shows under the same change. This gap indicates that the sustained gradient signal maintained by LSM depends more strongly on learning-rate decay.

Table S13: LSM convergence behavior under different learning rate schedules on the CCLD dataset

Configuration	LR Schedule	mAP@50	mAP@50:95	Final Train Loss	Loss Std (last 50 ep)
CIoU	Fixed (η=1e-3)	76.40	62.10	0.0342	0.0018
CIoU	Cosine Annealing	77.00	62.70	0.0285	0.0009
ABD-IoU	Fixed (η=1e-3)	77.20	63.50	0.0398	0.0052
ABD-IoU	Cosine Annealing	78.50	65.00	0.0271	0.0008

Loss curves (Figure S4) reveal the root cause of this difference. Under a fixed learning rate, ABD-IoU exhibits a loss standard deviation of 0.0052 over the final 50 epochs—far exceeding the 0.0008 observed with cosine annealing—and its final training loss is actually higher than the CIoU baseline. This pattern shows that the LSM non-zero gradient, without learning-rate decay to constrain it, induces persistent parameter oscillation. Cosine annealing gradually reduces the learning rate toward zero in later epochs, converting the gradient signal sustained by LSM into fine-grained refinement rather than destructive updates. The two mechanisms are therefore functionally coupled and must be deployed together.

S3.3 Comparison between HBB and OBB Frameworks

A comparison of the HBB and OBB paradigms on the CCLD dataset empirically validates the rationale for addressing anisotropic boundary regression within an HBB framework (Table S14). YOLOv8n-OBB improves mAP@50 by only 0.90% over its HBB counterpart. The limited gain stems from the absence of a stable principal orientation for cotton lesions: the statistics in Table 1 show that lesion major axes are distributed roughly uniformly across horizontal, vertical, and diagonal direction bands, preventing rotated boxes from reducing redundant area. The rotated NMS introduced by OBB also reduces T4 throughput by 31.6% and achieves only 52 FPS on Orin NX. At the annotation stage, the mean angular deviation between two annotators reaches 18.7°, making labeling consistency difficult to guarantee. The proposed method achieves 78.50% mAP@50

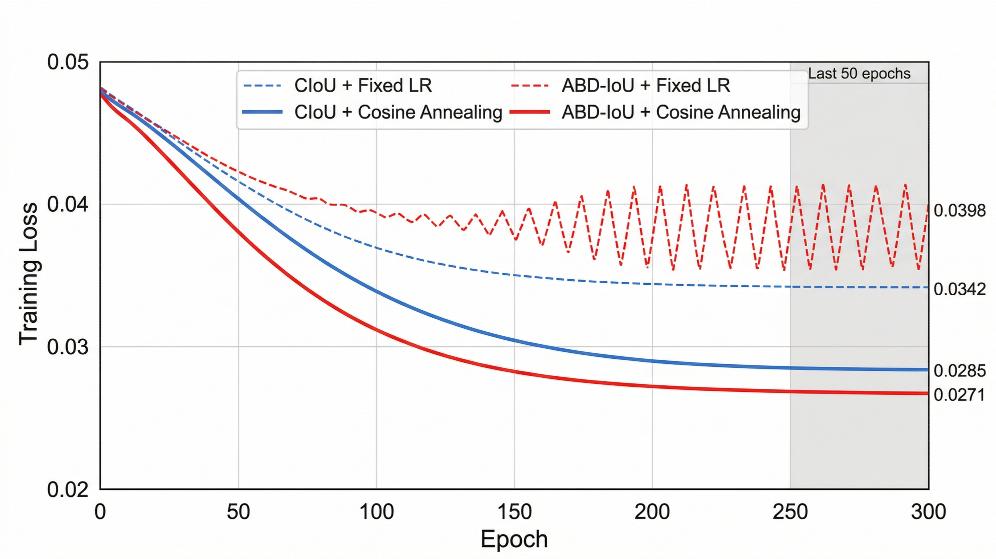

Figure S4: Training loss curves of CIoU and ABD-IoU under fixed learning rate ($\eta = 1e^{-3}$) and cosine annealing schedules on the CCLD dataset

within the HBB framework through four-boundary independent decoupling in ABD-IoU, surpassing the OBB approach by 6.40% while maintaining 72 FPS on Orin NX. It thus outperforms the OBB route along all three dimensions: accuracy, speed, and annotation feasibility.

Table S14: Comparison of horizontal bounding box (HBB) and oriented bounding box (OBB) frameworks on the CCLD dataset

Method	Framework	Params(M)	GFLOPs	mAP@50	mAP@50:95	FPS(T4)	FPS (Orin NX)
YOLOv8n	HBB	3.01	8.10	71.20	59.80	228	—
YOLOv8n-OBB	OBB	3.18	8.60	72.10	60.40	156	52
YOLOv11n	HBB	2.60	6.30	73.70	62.30	218	78
**Ours (Full)**	HBB	2.73	6.36	78.50	65.00	202	72

S3.4 Comparison of Loss Functions

Replacing only the loss function within the full model framework, we compare ABD-IoU against nine mainstream IoU losses (Table S15). From IoU to CIoU, progressive decoupling of penalty terms steadily raises mAP@50:95 from 61.80% to 62.70%. Beyond CIoU, improvements saturate and performance diverges across losses. EIoU surpasses SIoU on mAP@50 (77.20% vs. 77.10%), yet its APS falls below that of CIoU, indicating that width–height decoupling alone still provides insufficiently fine-grained gradient signals for small objects. Focal-EIoU achieves 63.00% mAP@50:95, lower than the unweighted SIoU at 63.10%, suggesting that Focal weighting suppresses gradients for moderately difficult samples—a negative side effect on a medium-scale dataset. NWD, through its Wasserstein metric, attains the highest APS among all non-proposed losses at 34.30%, yet its APL of only 71.80% is among the lowest of all improved losses, reflecting an adverse effect on large-object regression. ABD-IoU achieves the best results across all six metrics, improving over CIoU by 2.30% on mAP@50:95 and 1.80% on mAP@75, confirming the synergistic benefit of four-boundary

independent decoupling and LSM for refining irregular lesion boundaries.

Table S15: Comparison of nine mainstream IoU-family loss functions on the CCLD dataset

Loss Function	mAP@50:95	mAP@50	mAP@75	AP_S	AP_M	AP_L
IoU	61.80	76.20	66.30	32.10	57.60	71.20
GIoU	62.20	76.50	66.80	32.40	57.90	71.50
DIoU	62.50	76.80	67.10	32.80	58.10	71.80
CIoU	62.70	77.00	67.40	33.20	58.40	72.10
EIoU	62.90	77.20	67.60	33.00	58.60	72.30
SIoU	63.10	77.10	67.70	33.60	58.70	72.30
Focal-EIoU	63.00	77.40	67.80	33.80	58.80	72.40
Wise-IoU	63.20	77.50	67.90	33.50	58.80	72.50
NWD	63.30	77.30	67.50	34.30	58.50	71.80
**ABD-IoU (Ours)**	**65.00**	**78.50**	**69.20**	**35.10**	**60.20**	**73.80**

S3.5 Cross-Dataset Progressive Ablation Study

Reproducing the progressive ablation experiments from the CCLD dataset on the PlantDoc and RWD datasets verifies the independent contribution of each module and cross-module synergy in cross-domain settings (Table S16). On PlantDoc, the ranking of individual module gains is consistent with the pattern observed on CCLD, indicating that the functional role of each module is preserved across data domains. The combined AMCA + DSBT configuration yields a 2.20% gain, exceeding the sum of individual contributions by 0.50%—qualitatively consistent with the super-linear synergy observed on CCLD. The full model achieves 60.80% mAP@50 on PlantDoc, surpassing the baseline by 4.50%.

Table S16: Cross-dataset progressive ablation study on PlantDoc and RWD, replicating the CCLD ablation protocol (Table 2) to verify module-level generalization

Dataset	Baseline & Components					Detection Performance							Δ
	YOLOv11n	AMCA	DSBT	MFS-FPN	ABD-IoU	P	R	mAP@50	mAP@50:95	Param (M)	GFLOPs	Δ@50	FPS
Plant Doc	√	×	×	×	×	87.20	72.50	56.30	35.70	2.60	6.30	—	218
	√	√	×	×	×	85.80	74.10	57.20	36.10	2.35	6.20	+0.90	215
	√	×	√	×	×	84.60	73.80	57.10	36.00	2.70	6.31	+0.80	210
	√	×	×	√	×	86.50	73.20	57.40	36.40	2.61	6.33	+1.10	208
	√	×	×	×	√	87.40	72.80	56.80	36.10	2.60	6.30	+0.50	216
	√	√	√	×	×	86.30	74.50	58.50	36.80	2.62	6.25	+2.20	211
	√	×	√	√	×	86.80	74.20	59.10	37.40	2.63	6.24	+2.80	207
	√	×	×	√	√	85.50	74.60	59.30	37.20	2.72	6.34	+3.00	204
	√	√	√	√	√	87.50	75.20	60.80	38.90	2.73	6.36	+4.50	202
RWD	√	×	×	×	×	90.10	78.30	71.10	44.60	2.60	6.30	—	218
	√	√	×	×	×	88.70	80.20	72.40	45.20	2.35	6.20	+1.30	215
	√	×	√	×	×	87.90	79.80	72.30	45.00	2.70	6.31	+1.20	210
	√	×	×	√	×	89.30	79.50	72.60	45.50	2.61	6.33	+1.50	208
	√	×	×	×	√	89.10	80.60	74.20	45.10	2.60	6.30	+0.60	216
	√	√	√	×	×	89.50	80.60	74.80	47.20	2.63	6.24	+3.70	211
	√	×	√	√	×	88.40	80.80	75.10	47.00	2.72	6.34	+4.00	204
	√	×	×	√	√	89.70	80.50	76.10	48.80	2.73	6.36	+5.00	205
	√	√	√	√	√	90.40	81.30	76.80	49.50	2.73	6.36	+5.70	202

On RWD, the absolute gains of all modules are generally higher than on PlantDoc. MFS-

FPN alone contributes 1.50% and AMCA contributes 1.30%, reflecting the strong adaptability of anisotropic receptive fields and frequency-aware gating to lesions that spread along the parallel venation structure of narrow wheat leaves. The full model reaches 76.80% mAP@50 on RWD, exceeding the baseline by 5.70%—a gain larger than those on both CCLD and PlantDoc, further demonstrating that the module designs deliver consistent improvements across different crop morphologies.

S3.6 Cross-Dataset Grad-CAM Visualization of Ablation Generalization

Figure S5 presents Grad-CAM visualizations that trace how each module progressively sharpens feature focus during cross-domain evaluation (PlantDoc and RWD). Without fine-tuning, the baseline model diffuses activation broadly across unseen background textures. Adding AMCA and DSBT markedly increases the sensitivity of the network to high-frequency pathological features and structural edges while beginning to suppress false activations. Once MFS-FPN is integrated, spatially equivalent frequency decoupling strips away residual low-frequency background interference, and the heatmap of the full model converges tightly onto actual lesion regions. Together, these visualizations confirm that the morphology–frequency synergy strategy effectively suppresses domain-shift noise.

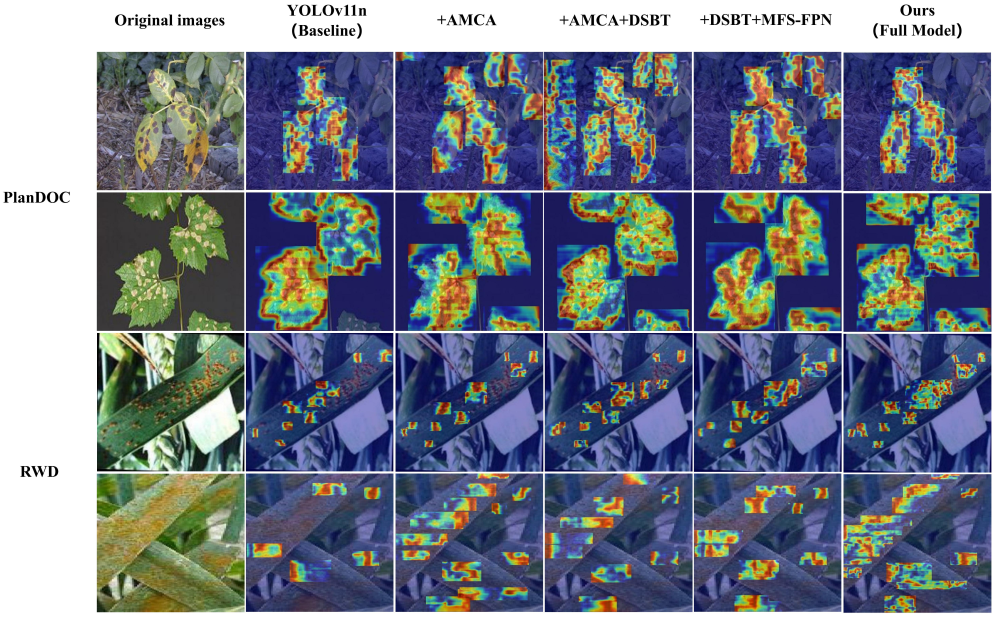

Figure S5: Grad-CAM heatmap visualization of the cross-dataset ablation generalization study

S3.7 Cross-Dataset Detection Visualization

Figure S6 compares qualitative detection results between the proposed method and the baseline across cross-domain scenarios. On complex CCLD scenes, the baseline is prone to false or missed detections caused by homogeneous background interference such as withered leaves. Under the larger domain shifts of PlantDoc and RWD, the baseline—heavily reliant on texture priors from the source domain—produces extensive missed detections against unseen backgrounds (red dashed boxes). The full model, by contrast, achieves precise bounding-box localization across all three datasets, recalling faint lesions without notable false positives (yellow dashed boxes). These results

further demonstrate that directional feature extraction combined with spatial-domain high-/low-frequency separation maintains consistent detection accuracy advantages over the baseline across different crops.

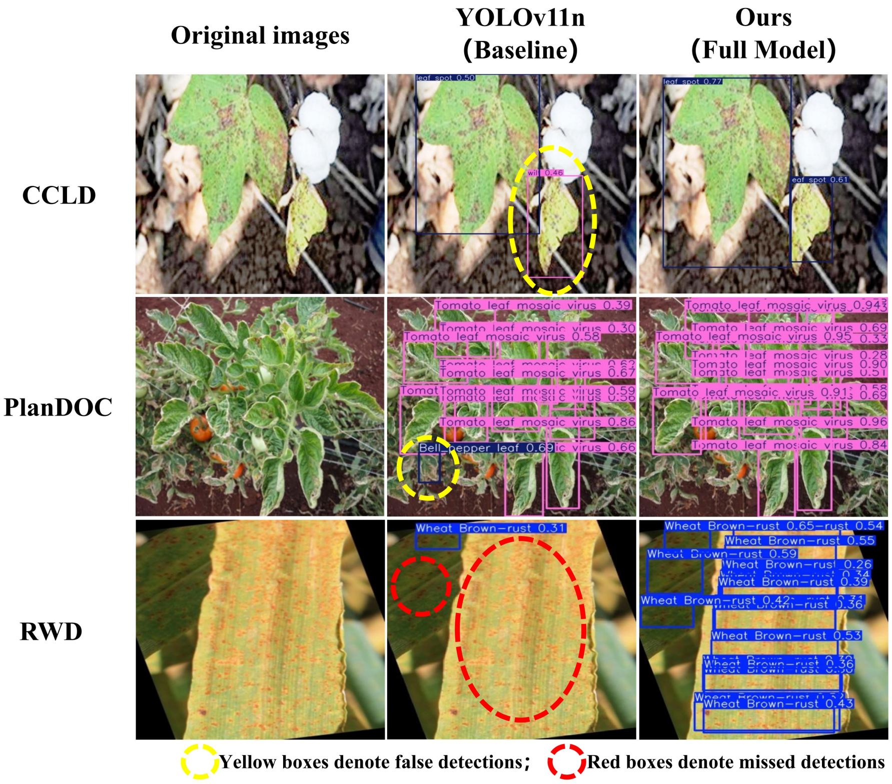

Figure S6: Cross-dataset detection visualization on PlantDoc and RWD

S3.8 Failure Case Analysis

Figure S7 presents typical failure cases of the proposed method under extremely challenging conditions. False detections occur primarily where leaves exhibit severe mechanical damage, which is highly isomorphic to pathological necrotic tissue in both RGB space and high-frequency response, creating visual ambiguity that the model cannot resolve. In dense canopy-overlap zones, natural high-frequency edges of certain healthy leaves are also occasionally misclassified by DSBT as disease boundaries. Missed detections are concentrated on latent-stage infections or tiny lesions under heavy shadow, where the high-frequency contrast of the target is almost entirely lost. Together, these cases delineate the perceptual upper bound of purely morphological and RGB frequency-domain feature learning when facing extreme physical interference and invisible-spectrum limitations.

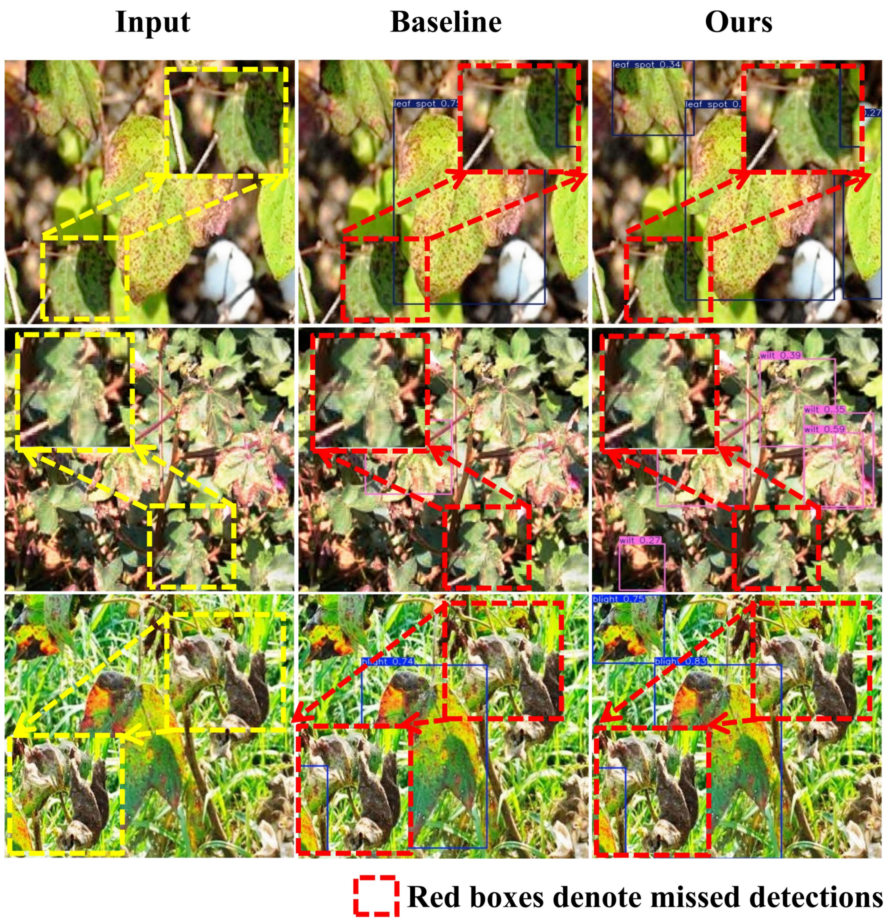

Figure S7: Failure case analysis
